# The role of pollination in controlling *Ginkgo biloba* ovule development

**DOI:** 10.1111/nph.17753

**Published:** 2021-10-11

**Authors:** Greta D’Apice, Silvia Moschin, Fabrizio Araniti, Sebastiano Nigris, Maurizio Di Marzo, Antonella Muto, Camilla Banfi, Leonardo Bruno, Lucia Colombo, Barbara Baldan

**Affiliations:** ^1^ Botanical Garden University of Padova Padua 25123 Italy; ^2^ Department of Biology University of Padova Padua 35121 Italy; ^3^ Department of Agricultural and Environmental Sciences University of Milano Milan 20133 Italy; ^4^ Department of Biosciences University of Milano Milan 20133 Italy; ^5^ Department of Biology, Ecology and Earth Sciences (DiBEST) University of Calabria Arcavacata of Rende CS 87036 Italy

**Keywords:** *Ginkgo biloba*, gymnosperms, metabolomics, ovule development, ovule morphology, pollination, transcriptomics

## Abstract

- Generally, in gymnosperms, pollination and fertilization events are temporally separated and the developmental processes leading the switch from ovule integument into seed coat are still unknown. The single ovule integument of *Ginkgo biloba* acquires the typical characteristics of the seed coat long before the fertilization event. In this study, we investigated whether pollination triggers the transformation of the ovule integument into the seed coat.
- Transcriptomics and metabolomics analyses performed on ovules just prior and after pollination lead to the identification of changes occurring in *Ginkgo* ovules during this specific time.
- A morphological atlas describing the developmental stages of ovule development is presented. The metabolic pathways involved in the lignin biosynthesis and in the production of fatty acids are activated upon pollination, suggesting that the ovule integument starts its differentiation into a seed coat before the fertilization.
- Omics analyses allowed an accurate description of the main changes that occur in *Ginkgo* ovules during the pollination time frame, suggesting the crucial role of the pollen arrival on the progression of ovule development.

## Introduction

The origin of seed traces back to the Devonian period, *c*. 360 million years ago (Ma) and represents a key innovation at the basis of the reproductive success of seed plants (Gerrienne *et al*., 2004; Gerrienne & Meyer‐Berthaud, 2007; Prestianni & Gerrienne, 2010; Meade *et al*., 2020). Seeds are formed upon single or double fertilization occurring inside ovules, therefore representing the endpoint of the ovule ontogeny (Meade *et al*., 2020).

Most Devonian and early Carboniferous ovule fossils showed a lobate integument with partially fused or free lobes surrounding the nucellus and a cupule. The nucellus, typically, developed a single functional megaspore (FM) and an apical modification for pollen reception (hydrasperman‐type pollen chamber) (Gerrienne *et al*., 2004; Prestianni & Gerrienne, 2010; Meade *et al*., 2020). In the most primitive ovules the integuments surrounded the nucellus only at the base. The complete enclosure of the nucellus evolved gradually to provide increased protection to the female gametophyte and the embryo (Gasser & Skinner, 2019; Meade *et al*., 2020). The single integument of gymnosperm ovules may be considered homologous to the inner integument of angiosperm ovules (Singh, 1978; Doyle, 2006). Instead, the outer integument of angiosperm ovules might derive from an already existing structure, such as, for instance, the cupule (Doyle, 2006, 2008; Endress & Doyle, 2009; Endress, 2011).

The main molecular mechanisms and genes responsible for ovule development have been largely studied in *Arabidopsis thaliana*, in which the mature ovule is composed of the female gametophyte surrounded by two integuments and a funiculus, as in the majority of angiosperms (Cucinotta *et al*., 2014; Barro‐Trastoy *et al*., 2020). In angiosperms, the pollination and fertilization events are temporally close: the male gametophyte (pollen grain) germinates, giving rise to the pollen tube, which ensures the delivery of the male gametes to the female gametophyte in order to form a seed (Figueiredo *et al*., 2016). Studies in the model plant Arabidopsis indicated that upon fertilization a signalling pathway becomes active in the endosperm and triggers the downstream processes responsible for the transformation of the ovule integuments into the seed coat (Figueiredo & Köhler, 2014). Recently, it has been demonstrated that the seed coat development process is activated by the fertilization of the central cell within the female gametophyte (Figueiredo & Köhler, 2018). The postfertilization production of auxin in the endosperm seems to be required for downregulation of the Polycomb Repressing Complex 2 (PRC2) gene expression, thus activating the downstream pathways, such as gibberellin (GA) biosynthesis and the accumulation of flavonoids needed for seed coat differentiation (Figueiredo & Köhler, 2018). By contrast, in gymnosperms, the processes that trigger the switch from ovule integument into seed coat are still not known. In addition, in *Ginkgo biloba* – and in most gymnosperms – pollination and fertilization are temporally separated events, suggesting that the pollen arrival could be the crucial event that leads to the further progression of the ovule development and the subsequent transformation of the ovule integument into the seed coat.

*Ginkgo biloba* was chosen for this study because of its interesting isolated phylogenetical position and the availability of its genome. There are some studies on *Ginkgo* ovule morphology available – the first morphological works date back to Carothers (1907), Sprecher (1907), and Takaso (1980) – but a comprehensive study of all developmental stages, to refer to in future studies, has not been provided so far. Therefore, in this paper we have analysed all *G. biloba* ovule developmental stages in detail with the aim of providing an atlas that accurately describes the morphological stages from ovule initiation to fertilization. This morphological analysis has been accompanied by transcriptomics and metabolomics analyses performed on ovules collected in a narrow time window of the whole course of the ovule development; we have, indeed, focused on the event of pollination. Omics analyses were performed before and after the pollination event (on stages 7 and 8, the latter divided into four chronological substages (8.1, 8.2, 8.3, and 8.4) of the ovule development) in order to determine whether pollen arrival is decisive in driving the processes of ovule integument transformation into seed coat. Transcriptomics goals were to identify regulative pathways altered following pollination, and to compare ‘switch genes’ that are activated following fertilization in Arabidopsis with genes that are differently regulated following pollination in *Ginkgo*. Metabolomics analyses aimed to highlight the main metabolic pathways that change following the pollination event in *Ginkgo* ovules. The combination of both metabolomics and transcriptomics analyses allowed us to describe the main metabolic and regulatory changes that occur in *Ginkgo* ovules after pollination.

## Materials and Methods

### Plant material

*Ginkgo biloba* ovules were collected from two centuries‐old trees at the Botanical Garden of Padua, Italy, which were named GA and GN for the purpose of this study. For the morphological studies, buds and pools of ovules were collected weekly from October 2019 until October 2020. Molecular and metabolomics analyses were conducted on ovules collected exclusively during the pollination time frame.

### Fresh sample observations

Pictures of fresh samples were acquired with a digital camera equipped with a macro lens. Fresh dissected samples were observed through a stereomicroscope (Leica EZ4W; Leica Microsystems, Wetzlar, Germany) equipped with a digital camera.

### Scanning electron microscopy observations

Samples were fixed in a 4% paraformaldehyde solution in 1× phosphate buffered saline (PBS; 10× PBS: 1.3 M sodium chloride, 70 mM disodium phosphate, 30 mM sodium dihydrogen phosphate; pH 7.0) with mild vacuum infiltration, and maintained in fixative overnight at 4°C. Then, samples were washed with 1× PBS (two times 30 min each wash) and dehydrated using an ethanol series (30%, 50%, 70%, 85%) with 1 h for each step, followed by 95% ethanol overnight, and finally two 100% ethanol stages for 30 min each. After dehydration, samples were treated with CO_2_ to reach the critical‐point drying and then coated with a layer of gold. Samples were observed under a Zeiss SUPRA 35VP scanning electron microscope equipped with an Oxford Instruments INCA X‐Sight detector (Oxford Instruments, Abingdon, UK).

### Paraffin embedding and sample section observations

Tissue fixation and dehydration protocols are the same as described earlier for the observation with the scanning electron microscopy. After the last dehydration step, ethanol 100% was gradually replaced with a xylene series (1 : 3; 1 : 1; 3 : 1; 4 : 0; 4 : 0 xylene : ethanol, for 1 h each). Xylene was gradually replaced by Paraplast Plus (Leica) – as described by Douglas *et al*. (2007). *Ginkgo* samples were embedded within steel base moulds and maintained in plastic embedding rings at 4°C until they were processed. Sections of 8–10 µm were cut on a Leica RM 2125 RTS microtome. Slides were deparaffinized and rehydrated to be stained with 1% (w/v) toluidine blue. Slides were observed with a Leica DM500 optical microscope.

### RNA extraction and sequencing

Pools of *G. biloba* ovules at five developmental stages around the pollination time were collected from the two centuries‐old trees GA and GN. GA and GN plants were considered to be two biological replicates for the purpose of RNA sequencing (RNA‐seq) analysis. GA and GN are male plants that have been grafted with female branches; therefore, male and female strobili are coordinated as they are under the same environmental conditions and in close proximity. This ensured an efficient and simultaneous pollination of most of the ovules collected during the sampling. The five sequenced stages are as follows: stage 7 (the pre‐pollination stage); substage 8.1 (the pollination drop substage); and substages 8.2, 8.3, and 8.4 (three postpollination drop substages 4, 6, and 8 d, respectively, after the emission of the pollination drop). Substages 8.1, 8.2, 8.3, and 8.4 are chronological substages of the eighth stage (Table 1), arbitrarily designated for the purpose of the study as at these times the ovules are not distinguishable from a purely morphological point of view.

**Table 1:** Stages of *Ginkgo biloba* ovule development.

Stage		Description	Ovule size (diameter)	Stage duration	Supporting literature	
					Corresponding stages in Douglas *et al*. (2007)	Corresponding stages in Jin *et al*. (2012b)
1		Emergence of the ovule primordia		Throughout summer and the following winter the ovules remain enclosed within buds	1	1
2		Differentiation of ovule primordia	< 500 µm		2	2
3		Nucellus and integument differentiation	*c*. 500 µm	*c*. 40 d before the pollination event^1^	3	3
4		Integument growth begins to enclose the nucellus	*c*. 500 µm		3	4
5		Integument has completely enclosed the nucellus. Ovular collar differentiation	> 500 µm	*c*. 20 d before the pollination event	4–5	5
6		Meiosis of the MMC and subsequent mitosis of the functional megaspore. Development of the female gametophyte starts	*c*. 1 mm		6	
7		**Pre‐pollination stage**. Micropyle, micropyle canal, and pollen chamber are completely formed	*c*. 1.5 mm	About a week before the pollination event	7	6
8	8.1	**Pollination drop substage**	*c*. 2 mm	4/5 d in which the pollination drop is emitted	7	6
	8.2	**Postpollination drop substages**		4 d after substage 8.1		
	8.3			6 d after substage 8.1		
	8.4			8 d after substage 8.1		
9		Female gametophyte growing. Integument layers are becoming distinguishable	2 mm to *c*. 15 mm	After the pollination event, the coenocytic growth of the female gametophyte takes *c*. 2 months		
10		Cellularization of the female gametophyte	*c*. 15 mm to *c*. 25 mm	*c*. 50–70 d after pollination		
11		Sclerotesta lignification	*c*. 25 mm to *c*. 28 mm	Visible after *c*. 75–90 d after pollination		
12		Archegonia completely formed	Sarcotesta softening^2^	*c*. 120–130 d after pollination		
13		Formed embryo	Sarcotesta softening^2^	*c*. 150 d after the pollination event		

MMC, megaspore mother cell.

Bold indicates transcriptomics and metabolomics analyses were performed on these stages.

^1^ Ovules at different stages of development can be found within the same bud.

^2^ Ovules/seeds stopped increasing in size, and the softening of sarcotesta causes an apparent decrease in width.

Total RNA was extracted using the protocol described by Chang *et al*. (1993). RNA samples were quantified using an Implen NanoPhotometer® (Implen GmbH, Munich, Germany) and RNA quality was assessed calculating the RNA integrity number (Agilent 2100 Bioanalyzer, Agilent Technologies, Santa Clara, CA, USA). PE 2 × 150 bp RNA‐seq was performed by Novogene (HK) using an Illumina NovaSeq 6000 sequencer (Illumina, San Diego, CA, USA). Raw reads were processed by Trimmomatic (Bolger *et al*., 2014) performing reads pairing, quality trimming and Illumina adapter removal. Gene expression was calculated on the processed reads by Salmon (Patro *et al*., 2017) as transcripts per kilobase per million of reads (TPM). RNA‐seq expression data were validated and reported as a ratio between TPM of the gene of interest (GOI) and TPM of the internal reference gene *Elongation Factor 2* (*EF2*; *Ginkgo* coding sequence (CDS): Gb_02896). Pearson correlation between the results of GA and GN samples was calculated by Novogene (HK) using the pipeline for RNA‐seq analysis of the ENCODE project. Expression levels of some selected genes were directly compared to further confirm replicate consistency. Differentially expressed genes (DEGs) were obtained with DESeq2 (Love *et al*., 2014). All RNA‐seq files are available in the National Center for Biotechnology Information (NCBI) database (BioProject ID code PRJNA700482).

### Genome annotation

*Ginkgo* CDSs together with the genome draft in the GigaScience Database (Guan *et al*., 2016) were annotated in this study using the Diamond software (Buchfink *et al*., 2015) using TrEMBL and Swiss‐Prot protein databases as reference. The annotation is available in the Supporting Information Table S1.

### Reverse transcription quantitative real‐time PCR analysis

Total RNA was retro‐transcribed from the same samples used for RNA‐seq analysis using the iScript™ gDNA Clear cDNA Synthesis kit (Bio‐Rad). The reverse transcription quantitative real‐time PCR (RT‐qPCR) experiment was performed in technical triplicate of the two biological replicates (GA and GN plants) in a Bio‐Rad iCycler iQ Optical System using the iTaq Universal SYBR Green Supermix, Bio‐Rad (http://www.bio‐rad.com). *EF2* (*Ginkgo* CDS: Gb_02896) was chosen as internal reference gene. The quantification of expression has been calculated using the $2^{‐\Delta \Delta C_{t}}$ method (Livak & Schmittgen, 2001; Pfaffl, 2001). The Bio‐Rad Software CFX Maestro™ was used to analyse data. The primers used are listed in Table S2.

### GC–MS‐driven untargeted metabolomics analysis

#### Samples extraction, derivatization, and analytical conditions

*Ginkgo* ovules were collected at the pre‐pollination stage, at the pollination drop substage, and at the postpollination drop substage (stages 7, 8.1, and 8.4, respectively, in Table 1). Samples were snap‐frozen in liquid nitrogen, powdered, poured into 2 ml vials filled with argon, and stocked at −80°C until the day of analysis. Sample extraction, derivatization, and analysis were performed using a modified version (Landi *et al*., 2020) of the protocol proposed by Lisec *et al*. (2006) using ribitol as internal standard (0.2 mg ml^−1^). The analysis was carried out using a Thermo Fisher gas chromatography apparatus (G‐Trace 1310, Thermo Fisher, Waltham, MA, USA) equipped with a single quadrupole mass spectrometer (ISQ LT). A 1 μl volume for each sample and replicate was injected in splitless mode into a capillary column (MEGA‐5 MS, 30 m × 0.25 mm × 0.25 µm + 10 m of pre‐column) (MEGA Srl, Legnano, Italy) using helium (6.0) as carrier gas with a flow rate of 1 ml min^−1^. Injector and source temperatures were settled at 250°C and 260°C, respectively, and samples were analysed using the following programmed temperature: isothermal 5 min at 70°C followed by a 5°C min^−1^ ramp to 350°C and a final 5 min heating at 330°C. Mass spectra were recorded in electronic impact (EI) mode at 70 eV, scanning in the 40–600 *m*/*z* range, scan time 0.2 s. The mass spectrometric solvent delay was settled as 9 min. *n*‐Alkane standards (C10–C40 all even) and blank solvents were injected at scheduled intervals for instrumental performance, tentative identification, and monitoring of shifts in retention indices.

#### Analysis of metabolomics data

Raw GC–MS data were then analysed using the software MS‐Dial v.4.48 coupled with a purpose‐built EI spectra library. The software parameters used for data collection, peak detection, deconvolution, alignment, and filtering were as previously reported in Fausto *et al*. (2021). Data annotation was carried out in MS‐Dial using publicly available libraries. Identification of compounds was based on comparison of the mass spectral pattern with EI spectral libraries, such as MoNA (Mass Bank of North America, http://mona.fiehnlab.ucdavis.edu/), MassBank, and the mass spectra and retention time index spectral libraries from the Golm Metabolome Database (Horai *et al*., 2010). Metabolite annotation and assignment of the EI‐MS spectra were achieved following the guidelines for the metabolomics standards initiative for compounds identification; that is, level 2 (identification was based on a spectral database) and level 3 (only compound groups were known, i.e. specific ions and RT regions of metabolites) (Sansone *et al*., 2007).

Experiments were carried out using a completely randomized design with three technical replications for each developmental stage (*n* = 3). Metabolomics data were analysed using the software Metabo analyst 5.0 (Chong & Xia, 2020). Metabolomics data were normalized using the internal standard (ribitol 0.02 mg ml^−1^) based normalization functions in the MS‐Dial software. The internal standard normalized data set was transformed through ‘log_2_ normalization’ and Pareto scaled. The data were then classified through unsupervised multivariate principal component analysis (PCA). The output comprised score plots to visualize the contrast between different samples and loading plots to explain the cluster separation. Metabolite variations were presented as a heatmap reporting only the ANOVA significant features (see the Results section). Partial least‐squares discriminant analysis (PLS‐DA) was used to highlight differences among the metabolic phenotypes at three time‐points (stages 7, 8.1, and 8.4) and to identify the metabolites mainly involved in groups separation as well as their change in concentration along time. Successively, data were analysed through the univariate ANOVA using Fisher's *post hoc* least significant difference test (*P* ≤ 0.05 to highlight statistical differences among single metabolites and ovule developmental stage). A false discovery rate was applied to the nominal *P*‐values as control for false‐positive findings. Further, for classification and features selection, a random forest analysis was carried out (Notes S1; Breiman, 2001; Enot *et al*., 2006; Chen *et al*., 2013) using 500 trees and seven predictors. Features with the highest interest were then ranked by their contributions to classification accuracy (mean decrease accuracy).

Finally, to identify the metabolite coverage and the main altered pathways during the three ovule stages of growth, data were analysed using the MetaboAnalyst enrichment analysis and the pathway analysis tool MetPA (Xia & Wishart, 2011). See Notes S1 for a deeper understanding of the univariate and multivariate approach in metabolomics experiments, and see Saccenti *et al*. (2014) and Percival *et al*. (2020) for a detailed review on the joint application of univariate and multivariate analysis.

#### Metabolomics raw data sharing

The raw data sets and the metadata associated with the GC–MS‐based metabolomics analysis have been deposited at the Mendeley database (doi: 10.17632/k8hghnmnpg.1) and are freely available for download from https://data.mendeley.com/datasets/k8hghnmnpg/1.

## Results

### Ovule development in *Ginkgo*: a long story

To date, only partial descriptions limited to a few developmental stages of the *Ginkgo* ovule could be found in some of the studies published so far (Douglas *et al*., 2007; Jin *et al*., 2012b; Wang *et al*., 2014). Here, we document and describe 13 subsequent stages of ovule development discriminated by appreciable morphological changes in developing ovules. We have created for the first time a comprehensive morphological atlas about the *Ginkgo* ovule developmental stages, consistent with the already existing literature (Lee, 1955; Avanzi & Cionini, 1971; Douglas *et al*., 2007; Jin *et al*., 2012b; Wang *et al*., 2014).

**Fig. 1:**
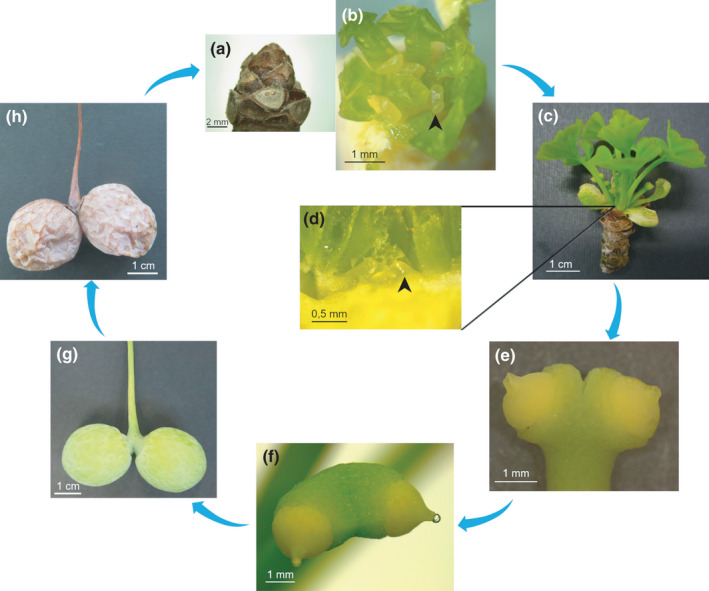
The entire development process of the ovule of *Ginkgo biloba* until seed maturation. Clockwise: (a) wintering bud; (b) opened wintering bud with ovules already present inside (black arrow‐head); (c) just opened bud with exposed leaves and ovules; (d) enlarged detail from (c): following year ovule primordia already recognizable under metabolically active leaves at the short‐shoot tip (black arrow‐head); Stage 1 of ovule development; (e) developing ovules; (f) developing ovules with pollination drops; (g) ovule development throughout the summer, until the fertilization takes place in late summer; (h) mature seeds.

*Ginkgo* ovule development has been thoroughly investigated from ovule initiation (Fig. 1a,b) to fertilization (Fig. 1g) and its consequent transformation into a seed (Fig. 1h). In early spring (at the latitude of Padua), *Ginkgo* buds open, exposing young leaves and female reproductive structures (Fig. 1c,e) that, after few weeks, are ready to be pollinated (Fig. 1f). At the same time, ovule primordia that will be exposed in the following spring are already forming on the same short‐shoot tip under leaves and recently blossomed ovules (stage 1; Fig. 1d). Thus, ovule primordia remain quiescent for almost a year. Once sprouted and successfully pollinated (Fig. 1f), ovules continue their process of development and maturation until the fertilization occurs after about 4 months (Fig. 1g).

The morphological steps of ovule development described in the following are summarized schematically in Table 1.

#### Ovule development within buds

**Fig. 2:**
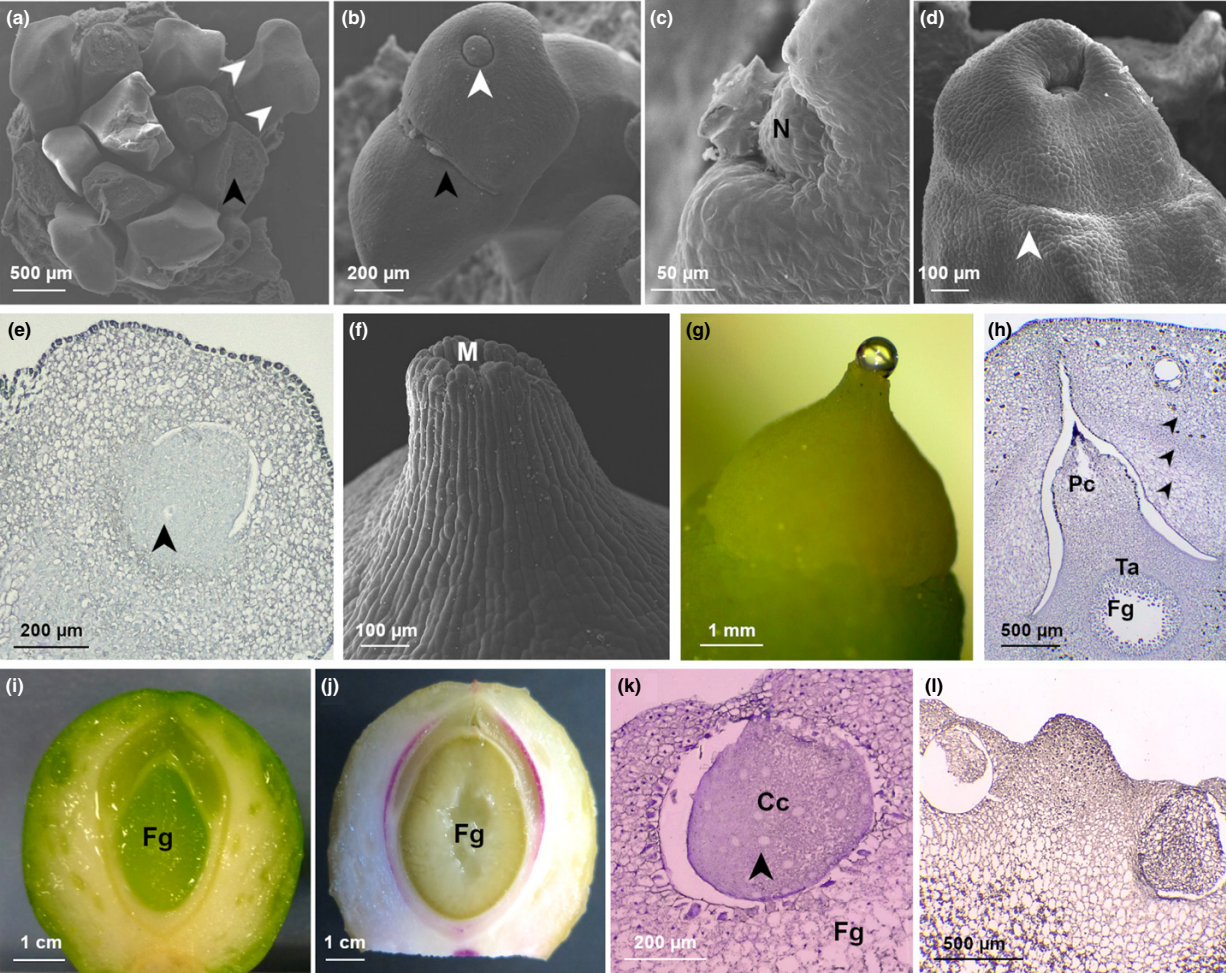
Stages of *Ginkgo biloba* ovule development. (a) Stage 2: differentiation of ovule primordia. Scanning electron microscopy (SEM) image of a bud deprived of leaves primordia (black arrowhead) showing developing ovules (white arrowheads show the two ovule primordia borne on a single stalk). (b) Stage 3: nucellus and integument differentiation. SEM image of ovules in which the forming nucellus is visible (white arrowhead); the sulcus, which separates the two ovules, is also shown (black arrowhead); polar view. (c) Stage 4: integument growth begins to enclose the nucellus. SEM image showing the detail of the ovule integument growing and flanking the nucellus, polar view. (d) Stage 5: integument has completely enclosed the nucellus; ovular collar differentiation. SEM image of an ovule showing the integument engulfing the underlying nucellus. Ovular collar is recognizable (white arrowhead). (e) Stage 6: meiosis of the megaspore mother cell (MMC) and subsequent mitosis of the functional megaspore; development of the female gametophyte starts. Longitudinal section of a paraffin‐embedded ovule showing the MMC (black arrowhead) in the centre of the nucellus. (f) Stage 7: pre‐pollination stage. Micropyle, micropyle canal, and pollen chamber are completely formed. SEM detail of the micropyle. (g) Stage 8: pollination stage. Pollination drop exposed. (h) Stage 9: female gametophyte growing; integument layers are becoming distinguishable. Longitudinal section of a paraffin‐embedded ovule showing the three layers of the developing integument that are differentiating (indicated by black arrowheads). (i) Stage 10: cellularization of the female gametophyte. Longitudinal section of a fresh ovule showing the central green and cellularized female gametophyte. (j) Stage 11: sclerotesta lignification. Section of a fresh ovule showing the lignified sclerotesta (in purple) stained with phloroglucinol. (k) Stage 12: archegonia completely formed. Longitudinal section of a paraffin‐embedded ovule showing the archegonium that contains the differentiated archegonial central cell. Shortly before fertilization the central cell will divide to originate the egg cell that will be fertilized. Black arrowhead indicates lipid inclusions. (l) Stage 13: formed embryo. Longitudinal section of a paraffin‐embedded seed in which the embryo is visible within the archegonium on the right. The unfertilized archegonium on the left is degenerating. Cc, central cell; Fg, female gametophyte; M, micropyle; N, nucellus; Pc, pollen chamber; Ta, tapetum.

Stage 1 of ovule development is the undifferentiated stage of the arising ovular primordia (Fig. 1d). Stage 2 is characterized by a typical bone‐shaped emergence of ovule primordia. Then, the two ovule primordia that dichotomize from a single stalk become recognizable because of the distal elongation of their two opposite ends (Fig. 2a). Ovules do not present subtending bracts, as the ovule‐bearing stalk emerges directly from the base of the leaf axil. The whole stalk, bearing the two ovules’ primordia at stage 2 of ovule development, measures *c*. 1 mm. Thereafter, at stage 3, ovules continue to grow, separating themselves until the dorsal groove between the two growing integuments becomes visible and clearly separates the two primordia (Fig. 2b). Margins of the forming integument grow radially, encircling the developing nucellus. At this stage, the nucellus is lens shaped and clearly visible in the distal pole of the ovule (Fig. 2b). At stage 4, the growing integument has thickened in the regions flanking the nucellus (Fig. 2c). At this stage, the histologically recognizable nucellus is *c*. 10 cells wide in diameter (Fig. S1a), and the primary sporogenous cells will differentiate from the hypodermal layer of the nucellus. Within the same bud, ovules at different stages of development can be found together (indicated by 1 in Table 1; i.e. stages 3 and 4). At stage 5 of the ovule development, the integument continues to grow over the nucellus, gradually overtopping it and finally covering it. The nucellus is then positioned at the centre of the ovule. At this stage, the sulcus separating the two ovules is visible, and the ovular collar begins to differentiate from the base of the integument (Fig. 2d).

#### Opening of the buds and preparation for pollen reception until pollination

At stage 6, the buds open, exposing ovules due to the rapid elongation of ovular stalks. Each ovule measures *c*. 1 mm in diameter. The visible megaspore mother cell (MMC) appears larger than the surrounding cells of the sporogenous tissue (Fig. 2e). Shortly after, the MMC undergoes meiosis, leading to the formation of the tetrad. Only the chalazal cell of the tetrad persists, forming the FM that undergoes several nuclear divisions leading to the formation of the female gametophyte. At the same time, cavitation of the pollen chamber takes place due to programmed cell death of apical nucellar cells (Jin *et al*., 2012a). The pollen chamber is the structure responsible for receiving the pollen grains where they can germinate. At stage 7, the growth of the integument leads to the complete formation of the micropyle and the micropylar canal (Figs 2f, S1b). The micropyle is distally oriented with respect to the bifurcation of the two ovules, and it connects the completely formed pollen chamber with the outer environment. The pollen chamber is teardrop shaped, with its opening oriented toward the micropyle (Fig. S1b). External cells of the micropyle are rectangular, elongated, and parallel oriented among themselves. Internally the micropyle canal is coated with epidermal cells. At stage 8, ovules are receptive for pollen. At pollination time (Fig. 2g), the pollen chamber has reached its largest volume. The developing female gametophyte remains within the enlarging megaspore membrane; thus, at the time of pollination, the embryo sac will be in an early coenocytic stage of development. The portion of the sporogenous tissue encircling the female gametophyte (the tapetum) is distinguishable from outer cells because its cells are larger and stain differently from external nucellar cells (Fig. S1c). The pollination drop, mainly produced by nucellar tissues in correspondence to the pollen chamber (Jin *et al*., 2012a), is extruded through the micropyle (Fig. 2g). For the purpose of this study, stage 8 was further divided into four substages: the pollination drop substage (substage 8.1 in Table 1) and three temporally close postpollination drop substages almost indistinguishable from a morphological point of view (substages 8.2, 8.3, and 8.4 in Table 1), corresponding, respectively, to 4 d, 6 d, and 8 d after the emission of the pollination drop. The pollination drop substage and the three postpollination drop substages have been included in the metabolomics and molecular analyses carried out in the proximity of the pollination event.

#### Ovule development after pollination until fertilization

At *c*. 1 wk after pollen is deposited in the pollen chamber it starts to germinate (Fig. S1d). The male gametophyte development proceeds within the ovule; in fact, it draws nourishment from the nucellus (haustorial male gametophyte). Its development will last until the moment of fertilization, when the spermatozoids will be released (Friedman, 1987; Friedman & Gifford, 1997).

After pollination, the colour of the ovules changes from yellow to green (Fig. 1f,g). At stage 9 (Fig. 2h), the micropyle opening is reduced, and nucellus cells surrounding the pollen chamber collapse inward, reducing its narrow opening until closing it. The opening is finally sealed by stacked debris of dead nucellar cells (Fig. 2h). The pollen tube consumes the nucellar tissue, which is thinning also because of the enlargement of the coenocytic female gametophyte, which generally forms > 1000 free nuclei before the cellularization process begins (Lee, 1955). During stage 9, the three differentiated layers of the single integument are becoming distinguishable (Figs 2h, S1e). The outer layer of the integument becomes the sarcotesta, made by wide and isodiametric cells; this layer is also dotted with calcium oxalates and mucilaginous canals. This layer will undertake a process of ripening, becoming fleshy at maturity, as occurs in the pericarp of proper fleshy fruits of angiosperms. Immediately below this outer layer, the cells that will form the sclerotesta are recognizable. These cells are smaller, thick walled, and constitute a portion of tissue with a greater cell density. Underneath this denser tissue, more loosely organized and elongated cells are recognizable, and these are the cells that will form the inner layer of the integument: the endotesta. At first it is soft and translucent, and then it differentiates to become a paper‐like layer (Fig. S1e). At the same time, tapetal cells encircling the female gametophyte are degenerating (Fig. S1f). The cellularization of the female gametophyte (stage 10) proceeds centripetally: cell walls are formed from the outer portion of the gametophyte, proceeding towards the centre (Fig. 2i). While the female gametophyte completes its cellularization, two distinct clusters of cells characterized by a smaller size and a denser protoplasm are gathering in its micropylar side. A single cell within the two clusters enlarges (generally two clusters are formed, but sometimes even more), and this is the archegonial initial cell, while adjacent cells encircle the enlarging one (Wang *et al*., 2014). The female gametophyte is green due to the presence of Chl (Fig. 2i). Stage 11 is characterized by the lignification of the sclerotesta, highlighted by staining with phloroglucinol in fresh ovules in Fig. 2(l). At this stage the inner endotesta is still fleshy, but in a short time it will differentiate, forming the thin papery layer between the female gametophyte and the sclerotesta. At stage 12, archegonia are completely formed (Fig. 2k) and, externally, the softening of sarcotesta causes an apparent decrease in width (indicated by 2 in Table 1). Archegonia are constituted by the neck cells, the central cell, and the cubic epithelial cells that delimit the archegonia forming the archegonial jacket (Wang *et al*., 2014). The portion of the female gametophyte in which archegonia are located stains differently with respect to the underpart of it. Indeed, cells appear more loosely organized, their walls are thinner, and they do not contain starch grains, unlike outer female gametophyte cells (Fig. S1g). Approaching the fertilization time (*c*. 135–145 d after the pollination), the central cell (Fig. 2k) divides to form the egg cell and a ventral canal cell that will degenerate soon, prior to the fertilization event (Wang *et al*., 2014). *Ginkgo* is a zoidogamous gymnosperm, meaning that spermatozoids swim toward the archegonium, which opens subsequently to the conformational changes of neck cells, allowing the fusion of the two gametes (Wang *et al*., 2014). At stage 13, the newly formed embryo is visible (Fig. 2l) and rapidly increases in dimensions; for a detailed description of the embryo development, see Feng *et al*. (2018).

### Ovule transcriptome analysis around pollination reveals the involvement of important metabolic pathways

Interestingly, *Ginkgo* ovule integument acquires the typical characteristics of the seed integument long before fertilization takes place. Indeed, before fertilization, the three‐layered integument differentiates: the inner endotesta thins out, the middle sclerotesta completes its lignification, and the outer sarcotesta thickens and begins to accumulate fatty acids. Since we wanted to test whether pollination is responsible for the switch from ovule into seed integument, a transcriptome analysis of ovules before and after pollination was performed. The five sequenced stages are: stage 7, substage 8.1, and substages 8.2, 8.3, and 8.4 (Table 1). Ovules were collected from female branches grafted on the two male individuals present at the Botanical Garden of Padua. Therefore, female branches are exposed to a large amount of pollen, which ensures a successful pollination for most of the ovules. A high‐throughput RNA‐seq (Illumina NovaSeq 6000) analysis was performed for each sequencing experiment. The correlation analysis (Fig. S2) indicates that the RNA‐seq results of the two sampled plants (GA and GN) correlate well and, therefore, that the two plants can be treated as biological replicates. Reproducibility between the two replicates is also shown in Fig. S3, where expression levels of some selected genes in the GA samples are compared with those in the GN samples.

Among the 10 sequenced samples we have obtained an average of 118 389 936 clean reads, of which 97% was mapped to the reference genome. TPM values were used to normalize gene expression levels.

**Fig. 3:**
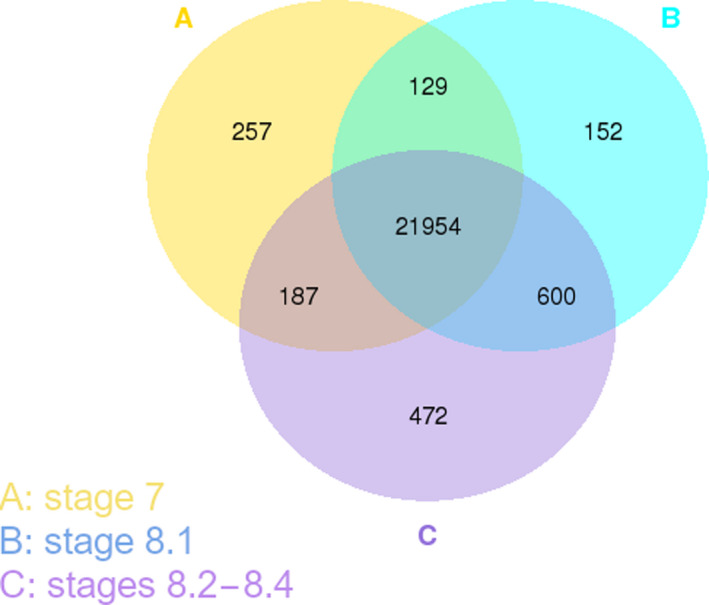
Coexpression Venn diagram from the RNA sequencing experiment performed on *Ginkgo biloba* ovules. Coexpression Venn diagram represents the significant Differentially expressed genes among the sequenced stages, considering the three postpollination drop substages (8.2, 8.3, and 8.4) as a unique postpollination stage.

To obtain an overview of the DEGs in the pre‐pollination, pollination drop, and postpollination drop stages, we considered substages 8.2, 8.3, and 8.4 as a unique postpollination drop stage (Fig. 3). This comparison evidenced that 257 genes are expressed only in stage 7, that 152 genes are specific for substage 8.1, and that 472 genes are expressed only in postpollination drop substages (Fig. 3).

**Fig. 4:**
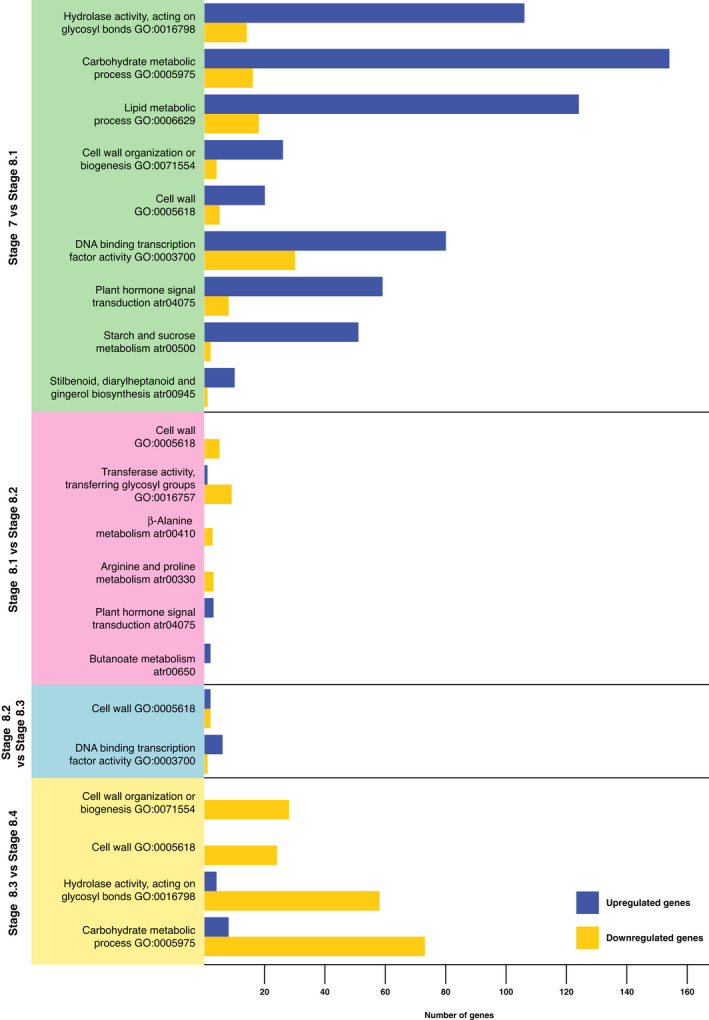
Diagram of the enriched pathways resulted from the RNA sequencing experiments performed on *Ginkgo biloba* ovules at five stages of development. The Gene Ontology (GO) and the Kyoto Encyclopedia of Genes and Genomes (KEGG) analyses indicate which pathways are significantly associated with differentially expressed genes. The graph shows the paired comparisons between the five sequenced substages, and significant GO and KEGG enriched pathways are reported for each comparison. The bar length indicates the number of up (blue bars) and downregulated (yellow bars) genes belonging to the respective pathways.

The Gene Ontology (GO) and Kyoto Encyclopedia of Genes and Genomes (KEGG) analyses allowed us to find out which biological functions or pathways are significantly associated with DEGs (GO and KEGG bar charts are provided, respectively, in Figs S4, S5). The results showed a more pronounced change in terms of DEGs between stage 7 and substage 8.1, and between substages 8.3 and 8.4 (Figs 4, S4, S5; Table 2). The GO analysis performed on comparison of stages 7 and 8.1 revealed that the ontological categories that include more DEGs are attributable to two macro‐categories of different cellular processes: the mobilization of energy resources, and the construction of cell walls. The comparison between substages 8.2 and 8.3 did not show significant DEG variation, whereas the GO analysis performed comparing substage 8.3 with substage 8.4 turned out to be opposite to what was observed comparing the pre‐pollination stage with the pollination drop substage. Indeed, the same pathways that are mostly upregulated comparing stage 7 and substage 8.1 are mostly downregulated comparing substages 8.3 and 8.4. The significant GO and KEGG enriched pathways resulting from the comparison between successive stages are reported in Table 2.

**Table 2:** Most significant Gene Ontology (GO) and Kyoto Encyclopedia of Genes and Genomes (KEGG) enriched pathways resulted from the comparisons between the five sequenced stages in the RNA sequencing experiment performed on *Ginkgo biloba* ovules during the pollination time.

Stage comparison	GO/KEGG enriched pathways	Upregulated genes	Downregulated genes
Stage 7 vs substage 8.1	Hydrolase activity, acting on glycosyl bonds GO:0016798	106	14
	Carbohydrate metabolic process GO:0005975	154	16
	Lipid metabolic process GO:0006629	124	18
	Cell wall organization or biogenesis GO:0071554	26	4
	Cell wall GO:0005618	20	5
	DNA binding transcription factor activity GO:0003700	80	30
	Plant hormone signal transduction atr04075	59	8
	Starch and sucrose metabolism atr00500	51	2
	Stilbenoid, diarylheptanoid, and gingerol biosynthesis atr00945	10	1
Substage 8.1 vs substage 8.2	Cell wall GO:0005618	0	5
	Transferase activity, transferring glycosyl groups GO:0016757	1	9
	β‐Alanine metabolism atr00410	0	3
	Arginine and proline metabolism atr00330	0	3
	Plant hormone signal transduction atr04075	3	0
	Butanoate metabolism atr00650	2	0
Substage 8.2 vs substage 8.3	Cell wall GO:0005618	2	2
	DNA binding transcription factor activity GO:0003700	6	1
Substage 8.3 vs substage 8.4	Cell wall organization or biogenesis GO:0071554	0	28
	Cell wall GO:0005618	0	24
	Hydrolase activity, acting on glycosyl bonds GO:0016798	4	58
	Carbohydrate metabolic process GO:0005975	8	73

In addition, we observed that some transcription factors belonging to the lignin regulatory network are differentially expressed when comparing stage 7 with substage 8.4 (Table 3), in accordance with the metabolomics analysis (see the following subsection). In particular, putative *Ginkgo* orthologues of Arabidopsis activators of the lignin biosynthesis pathway, such as *MYB61*, *MYB26*, *MYB46*, *MYB58*, and *NST2* (Zhao & Dixon, 2011), were upregulated.

**Table 3:** *Ginkgo biloba* transcription factors belonging to the lignin regulatory network that are differentially expressed between stages 7 and 8.4 of ovule development.

Arabidopsis orthologous genes	*Ginkgo* CDS	Substage 8.4 value	Stage 7 value	log_2_ (fold change)	*P*‐value	*P*‐adjust
*MYB63/MYB58*	Gb_13117	0.143429183	0.007802651	4.200473988	0.008482568	0.039487077
*MYB83/MYB61/MYB46/MYB26*	Gb_40065	11.82285882	4.138988129	1.514251186	1.01 × 10^−8^	2.95 × 10^−7^
	Gb_25814	3.014301106	0.259487982	3.538131659	6.22 × 10^−17^	5.13 × 10^−15^
	Gb_33428	13.50373396	4.983419618	1.43816343	1.04 × 10^−11^	4.92 × 10^−10^
*NST1/NST2/NST3*	Gb_01375	3.191131363	1.745278213	0.870700927	0.01060573	0.046704262
	Gb_32549	12.9950516	8.008710222	0.698343608	0.001507969	0.010148823

CDS, coding sequence.

On the other hand, the majority of the putative *Ginkgo* orthologues of Arabidopsis ‘switch genes’ – which are the genes that are activated or repressed upon fertilization and that belong to the gene regulatory network responsible for the switch from the ovule integuments into the seed coat (Figueiredo & Köhler, 2014, 2018; Figueiredo *et al*., 2016) – are not differentially expressed between pre‐pollination and postpollination drop substages, nor between pollination drop and postpollination drop substages (Table S3).

RNA‐seq expression levels were confirmed by RT‐qPCR performed on the same samples. RT‐qPCR analyses are, in most cases, consistent with the normalized expression levels of the gene of interest (TPM GOI/TPM *EF2*) provided by the RNA‐seq (Fig. S6). These transcription factors were chosen upon the role of the respective orthologues in Arabidopsis ovule development (Table S2).

### An untargeted metabolomics approach reveals a correspondence between molecular and metabolic pathways

**Fig. 5:**
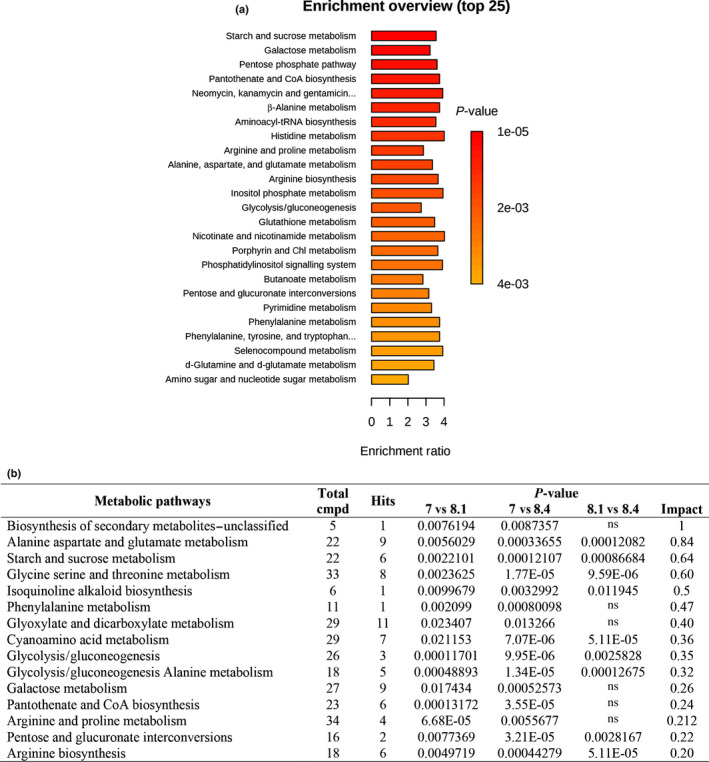
Kyoto Encyclopedia of Genes and Genomes‐based enrichment analysis of *Ginkgo biloba* ovules during the pollination phase. (a) Pathway enrichment analysis reveals different metabolic pathways enriched during different ovule stages (*P*‐value cut off ≤ 0.05). (b) The result from ‘Pathway Analysis’ carried out with the web‐based tool MetPA using the concentrations of metabolite identified in *Ginkgo* ovules during the pre‐pollination (stage 7), pollination drop (substage 8.1) and postpollination drop (substage 8.4) stages. Total cmpd, the total number of compounds in the pathway; Hits, the matched number from the uploaded data; Raw *P*, the original *P*‐value; Impact, the pathway impact value calculated from pathway topology analysis. The complete pathway analysis, including the full list of the pathways, the False discovery rate applied to the *P*‐value) and Holm adjustment (used to counteract the problem of multiple comparisons) are reported in Supporting Information Table S4. ns, no significant statistical difference.

The GC–MS‐driven analysis was performed on ovules collected at the pre‐pollination drop stage (stage 7), at the pollination drop substage (substage 8.1), and at the postpollination drop substage (substage 8.4). Among all three developmental stages, the metabolomics analysis allowed for an annotation and quantification of 201 metabolites and an extraction of 2832 unknown EI‐MS shared features. Both annotated and unknown metabolites, processed through MS‐Dial, have been reported as supplementary data (Table S4). The KEGG‐based enrichment analysis revealed an enrichment of the pathways for starch and sucrose metabolism, glycolysis/gluconeogenesis metabolism, galactose metabolism, pentose phosphate pathway, β‐alanine metabolism, arginine and proline metabolism, pantothenate and coenzyme A (CoA) biosynthesis, and phenylalanine metabolism, among others (Fig. 5a; Table S4). The pathway analysis, which combines enrichment and topology analysis, was carried out by comparing the different stages with each other (7 vs 8.1; 7 vs 8.4; 8.1 vs 8.4). The results indicated that 15 pathways, with an impact higher > 0.2, were significantly changed comparing the pre‐pollination stage with the pollination drop substage (7 vs 8.1) and the postpollination drop substage (7 vs 8.4) of ovule development. On the contrary, comparing substages 8.1 vs 8.4, only nine were significantly impacted (Fig. 5b; Table S4). ANOVA identified that 92 out of 201 metabolites were differentially produced among the three ovule developmental stages. These metabolites mainly belonged to chemical classes of the amino acids, organic acids, sugars and sugar alcohols, polyamines, fatty acids, and so on (Table S4). The majority of amino acids significantly increased in both stage 7 and substage 8.1. Only proline accumulated during stage 7. Successively, in substage 8.4, its content was restored to pre‐pollination levels. A similar trend was also observed in several sugars and sugar alcohols (Table S4). Whereas fructose, glucose, and trehalose accumulated during substage 8.1 and their content fell in the 8.4 samples, galactinol accumulated significantly only in substage 8.4.

**Fig. 6:**
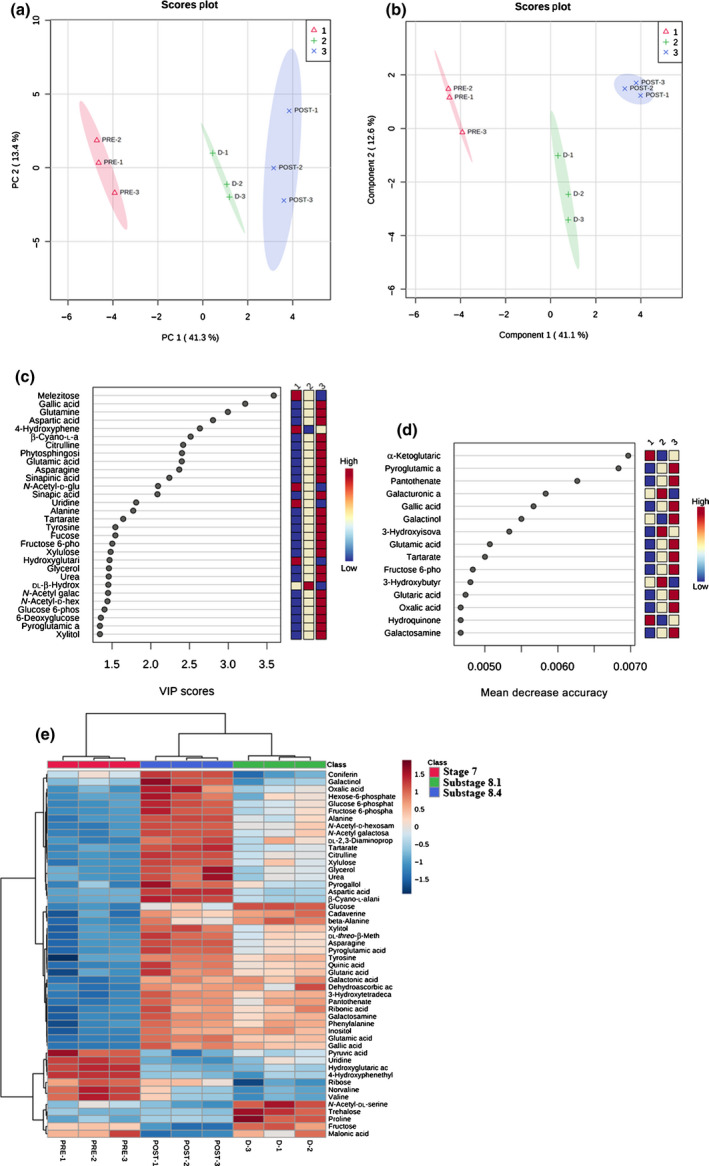
Discrimination through principal component analysis (PCA) and partial least square discriminant analysis (PLS‐DA) of *Ginkgo biloba* ovule samples in the three stages analysed based on metabolomics analysis. (a) PCA and (b) PLS‐DA showing score plots discriminating stage 7 (indicated in the legend with 1), substage 8.1 (indicated with 2), and substage 8.4 (indicated with 3) groups by virtue of the first two principal components (PCs). (c) Variable importance of projection (VIP) features for the groups from PLS‐DA analysis. (d) Random forest analysis displaying the mean decrease accuracies. (e) Overlay heatmap of the top 50 metabolites profiles (selected by ANOVA with *P* ≤ 0.05) in stage 7 (1), substage 8.1 (2), and substage 8.4 (3). PRE1–PRE3 are replicates of stage 7 (pre‐pollination stage); D1–D3 are replicates of substage 8.1 (pollination drop substage) and POST1–POST3 are replicates of substage 8.4 (postpollination‐drop substage). Each square represents the different stage's effect on every metabolite's relative abundance using a false‐colour scale. Colours dark red and blue indicate relative metabolite abundances, increased and decreased, respectively (*n* = 3).

Among the secondary metabolites involved in lignin biosynthesis (looking at the ANOVA, variable importance of projection (VIP) scores, and random forest outputs), quinic acid, sinapinic acid, sinapic acid, pantothenate, and phenylalanine significantly accumulated in both substage 8.1 and in substage 8.4, whereas coniferin, compared with stage 7, was significantly reduced in substage 8.1 and accumulated during substage 8.4. The unsupervised PCA was carried out on blank samples and on all three sample groups to demonstrate the system suitability (Fig. 6a,c). The PCA score plot, built on the first and second principal components (PC1 and PC2, respectively), revealed good discrimination of sample groups against blanks, highlighting model robustness (Fig. 6). The components used separated all three stages with no outliers (Fig. 6), indicating that our metabolomics analysis was reliable and could sufficiently reflect the metabolic profile changes of the ovule. Both unsupervised PCA analysis (Fig. 6a) and supervised PLS‐DA conducted on annotated metabolites (Fig. 6b) demonstrated group separation, with the first two PCs explaining 54.7% variance for PCA and 53.7% variance for PLS‐DA score plots. PLS‐DA‐derived VIP scores (built on the first 30 metabolites with a VIP score > 1.4) revealed melezitose, gallic acid, glutamine, and aspartic acid, among others, like the ones with the highest VIP scores for the three developmental stages (Fig. 6c). A random forest analysis – used to identify those metabolites that best classify the data into different groups and potential biomarkers of the phenomenon under study – revealed α‐ketoglutaric acid, pyroglutamic acid, pantothenate, galacturonic acid, gallic acid, and galactinol, among others, with the highest mean decrease accuracy (features ranked by their contributions to classification accuracy) for the three sample groups (Fig. 6d). Finally, cluster analysis on the top of the heatmap – reporting in a false colour scale the variation of metabolites’ concentrations for each sample and replicate – confirmed, at a lower level, total discrimination among all samples, whereas, at a higher level, substages 8.1 and 8.4 grouped together, suggesting a similarity between these two stages (Fig. 6e). This similarity is also revealed by the reduced number of pathways altered resulting from the pathway analysis (Fig. 5b).

## Discussion

The observation that *Ginkgo* female plants isolated from male plants (and, therefore, from the arrival of pollen) aborted all their ovules after the pollination drop emission (Friedman, 1987) supports the hypothesis of the crucial role of pollination in triggering the long differentiation process that modifies the ovule integument into a seed coat. To understand the molecular networks controlling the morphological changes observed, we performed transcriptomics and metabolomics analyses. GO and KEGG analyses showed that genes involved in the energetic metabolism are activated before the emission of the pollination drop, whereas genes belonging to the same pathways are downregulated after pollination. The untargeted metabolomics analysis strongly supported the transcriptomics data highlighting an alteration of starch and sucrose metabolism accompanied by an accumulation of proteinogenic amino acids, which are strictly interconnected (Münch, 1930; Hammes *et al*., 2006). In particular, proteinogenic amino acids were accumulating in the pollination and postpollination drop substages (8.1 and 8.4 substages, respectively), underlying the presence of an intense protein synthesis that is typical of growing and differentiating tissues. These amino acids are passively transported following the phloem bulk flow dictated by sucrose (transport and assimilation), representing the major osmolyte (Münch, 1930; Hammes *et al*., 2006). Starch and sucrose metabolism was one of the pathways affected most. Sucrose content was stable during all the stages of the ovule analysed, suggesting its cleavage to guarantee the phloem mass flow towards the growing tissues. In agreement with this hypothesis, an accumulation of glucose and fructose, the main products of sucrose breakdown mediated by invertases, was observed (Sturm & Tang, 1999). The possible degradation of sucrose was further suggested by increasing glucose‐6‐phosphate and fructose‐6‐phosphate content, which are the main products derived from glucose during glycolysis, guaranteeing a carbon flux towards the Krebs cycle. The modulated regulation of genes connected with sugars and amino acid metabolism is also consistent with studies on *Ginkgo* pollination drop composition, which demonstrated that the drop is mainly composed of sugars (at a high level) and amino acids (at a lower level) (Nepi *et al*., 2017; von Aderkas *et al*., 2018; Cheng *et al*., 2018; Prior *et al*., 2019; Lu *et al*., 2020).

*Ginkgo* ovules are characterized by an intense cell division during the pre‐pollination drop stage, which continues till the pollination drop substage is reached. Successively, pollinated ovules restart their growth, whereas unpollinated ovules abort and fall. Among the metabolites isolated in *Ginkgo* ovules, 4‐hydroxyphenethyl alcohol should be mentioned. It is present at high concentrations in the pre‐pollination stage, but its concentration drops down during the pollination drop substage and increases again after pollination. This molecule is known in the literature for its cytokinin‐like activity and its ability in promoting cell division (Serdyuk *et al*., 1995, 2000), and the observed fluctuation in its concentration could be involved in ovule growth before and after pollination.

Lastly, genes required for the defence against abiotic and biotic stresses are always expressed at high levels in the stages under consideration. This is probably because the micropyle and the pollination drop represent an open way and a vehicle for putative pathogens that could enter the ovule (von Aderkas *et al*., 2018; Cheng *et al*., 2018; Lu *et al*., 2020). Indeed, protection from potential bacterial and fungal pathogens is essential.

Beyond the descriptive aspect, the omics analyses on ovules before, during, and after pollination were fundamental also in order to acquire new knowledge of the pollination event in *G. biloba*, particularly with regard to whether it might be the trigger leading to the activation of downstream pathways responsible for the switch from ovule integument into seed coat. It is now well established that, in Arabidopsis, the seed coat initiation is dependent on the fertilization of the central cell and the subsequent formation of the triploid endosperm (Figueiredo *et al*., 2016). Following fertilization, auxin produced in the endosperm is transported to the integuments by PGP10, an ABCB‐type transporter that is controlled by the MADS‐box transcription factor AGL62 (Figueiredo *et al*., 2016). Once in the integuments, auxin removes the PRC2 block, thus allowing GA biosynthesis and the accumulation of flavonoids mediated by TRANSPARENT TESTA. Therefore, in Arabidopsis, during the switch from ovule integuments into seed coat, genes that are required for auxin and GA biosynthesis are upregulated together with genes involved in auxin transport, such as PGP and PIN1 transporters, while PRC2 and DELLA proteins are downregulated together with GA catabolism genes (Figueiredo *et al*., 2016; Figueiredo & Köhler, 2018). In *Ginkgo* ovules, most orthologous genes that in Arabidopsis are known to be involved in the switch from ovule into seed integuments are not differentially expressed before and after the pollination. A possible explanation can be found in the biological differences of the two plants. Indeed, pollination and fertilization are events that occur with very different timings in Arabidopsis and *Ginkgo*. In *Ginkgo*, the female gametophyte is haploid and the fertilization of the egg cell occurs months after pollination, once the ovule integument is already transformed, therefore showing the typical seed coat morphology. After fertilization, the seed coat of *Ginkgo* seeds does not undergo significant morphological modification except for the completion of the ripening process of the fleshy and outermost layer – the sarcotesta – necessary to enhance seed dispersal. Given these differences, it does not come as a surprise that in *Ginkgo* ovules the process of seed coat development, activated by pollen arrival, involves different genes.

In this study, we have highlighted some relevant changes occurring during pollination that can be interpreted as determinants for the transformation of ovule integument into seed coat in *G. biloba*. These results indicate that this switch is triggered by pollination, long before fertilization. Indeed, the seed coat of *G. biloba* is characterized by the differentiation in an outer and fleshy sarcotesta, characterized by the presence of fatty acids, among which butyric acid confers a foul odour to the seed, and in an inner sclerotesta, that constitutes the lignified layer protecting the inner female gametophyte and/or embryo (Singh *et al*., 2008; Nigris *et al*., 2021). During the switch from pre‐pollination to postpollination stages the metabolic pathways required for the biosynthesis of butanoate (the ester of butyric acid) and fatty acids are significantly enriched together with transcriptional activators of the lignin biosynthesis. The metabolomics and transcriptomics results obtained suggest that metabolic pathways involved in the seed coat formation are activated upon pollination. In fact, the pathway analysis pointed out that the phenylalanine metabolism, involved in lignin production, was one of the most impacted pathways. β‐Alanine and proline play a key role in lignin biosynthesis and regulation (Broeckling *et al*., 2005; Guan *et al*., 2019), besides being fundamental in the pollination process (Shivanna, 2003; Nepi, 2014; Nepi *et al*., 2017). The content of these amino acids confirmed the trend observed during the transcriptomics analysis. β‐Alanine, incorporated into pantothenate (significantly increased in substage 8.4), is a precursor of the acyl‐carrier protein CoA (White *et al*., 2001; Kupke *et al*., 2003), which also serves as a carrier for the lignin precursor sinapic acid (significantly accumulated in substage 8.4) and sinapinic acid (Yamauchi *et al*., 2002). Accumulation of metabolites such as quinic acid and the monolignol glucoside coniferin, both essential for lignin biosynthesis, suggested the activation of seed coat differentiation programmes upon pollination (Terashima *et al*., 2016; Volpi e Silva *et al*., 2019). In plants, quinic acid is conjugated with *trans*‐cinnamic acids for the biosynthesis of chlorogenic acids, which occurs principally via the shikimate shunt (the shikimate content was significantly increased in both 8.1 and 8.4 substages), which is also the major route towards the synthesis of lignin units (Volpi e Silva *et al*., 2019). Concerning coniferin, its role as lignin precursor in gymnosperms has been widely documented since monolignol 4‐*O*‐β‐d‐glucosides, such as syringin and coniferin, are considered as the primary transportation and/or storage forms of monolignols for lignification (Aoki *et al*., 2016; Terashima *et al*., 2016). However, a premature tissue lignification driven by the overproduction of lignin could strongly interfere with tissue growth and development. Recent studies have demonstrated that the proteinogenic amino acid proline can appropriately reduce lignin biosynthesis (Guan *et al*., 2019). Therefore, besides its implication as an osmoprotectant (Verbruggen & Hermans, 2008), in energy storage (Kishor *et al*., 2005), or its role in pollen germination (Shivanna, 2003), proline could play a pivotal role in modulating lignin production, suggesting that the accumulation observed in our experiments could serve also in maintaining lignin production under normal thresholds.

This study provides an accurate description of morphological steps that occur during ovule growth and development from ovule initiation to fertilization. This thorough structural characterization was prodromal to the omics analyses performed during the switch from pre‐pollination to postpollination stages. Furthermore, our results suggest that the process of seed coat development in *G. biloba* might be triggered by the pollination event (instead of fertilization as occurs in Arabidopsis), which leads to the formation of a ‘seed‐coat‐like postpollination ovule integument’ that will be already differentiated prior fertilization, displaying all the morphological characteristics of the mature seed coat.

The results obtained pave the way for understanding the molecular network controlling the development of ovule and seed structures in *Ginkgo* and for further investigations into the conservation of the ovule developmental programme in gymnosperms. Everything considered, these results do not allow us to state whether the mechanisms that drive the seed coat development are conserved or not across seed plants, as extant gymnosperms and angiosperms form two sister clades whose lineages separated *c*. 300 Ma (Becker *et al*., 2000); therefore, both groups experienced a long, independent evolutionary history. Further studies will be needed to better elucidate these questions.

## Author contributions

BB, LC, LB and FA conceived the study; GD'A, SM, SN, FA, AM, MDM and CB conducted the experiments; SN, GD'A, and SM analysed sequencing data; GD'A and SM wrote the first draft; SN, BB, LC and FA provided critical editing of the manuscript. GD'A and SM contributed equally to this work.

## Supporting information


**Fig. S1** *Ginkgo biloba* ovule development: supporting morphological analysis.
**Fig. S2** Correlation analysis between the two RNA‐seq experiments performed on the two plants of *Ginkgo biloba* (GA and GN) in the five sequenced stages.
**Fig. S3** Comparison of the expression levels (log_2_ TPM) of selected genes in the two plants of *Ginkgo biloba*.
**Fig. S4** Gene Ontology (GO) enrichment bar chart.
**Fig. S5** Kyoto Encyclopedia of Genes and Genomes (KEGG) enrichment bar chart.
**Fig. S6** Comparison between the RNA‐seq expression levels (blue line) and RT‐qPCR expression levels (orange line) performed on *Ginkgo biloba* ovules during the pollination phase.
**Notes S1** Deepening of univariate and multivariate approach in metabolomics experiments.
**Table S2** Primer sequences used to amplify *Ginkgo biloba* selected genes.

**Table S1** *Ginkgo biloba* genome annotation.

**Table S3** *Ginkgo biloba* orthologous genes of Arabidopsis ‘switch genes’.

**Table S4** GC‐MS‐driven untargeted metabolomics analysis data.
Please note: Wiley Blackwell are not responsible for the content or functionality of any Supporting Information supplied by the authors. Any queries (other than missing material) should be directed to the *New Phytologist* Central Office.

## Data Availability

The data that support the findings of this study are openly available in the NCBI database at http://www.ncbi.nlm.nih.gov/bioproject/700482, reference no. PRJNA700482, and in the Mendeley database at https://data.mendeley.com/datasets/k8hghnmnpg/1, reference no. 10.17632/k8hghnmnpg.1. Correspondence and requests for materials should be addressed to BB.

## References

[nph17753-bib-0001] von Aderkas P , Prior NA , Little SA . 2018. The evolution of sexual fluids in gymnosperms from pollination drops to nectar. Frontiers in Plant Science 9: e1844. 10.3389/fpls.2018.01844 PMC6305574 30619413

[nph17753-bib-0002] Aoki D , Hanaya Y , Akita T , Matsushita Y , Yoshida M , Kuroda K , Yagami S , Takama R , Fukushima K . 2016. Distribution of coniferin in freeze‐fixed stem of *Ginkgo biloba* L. by cryo‐TOF‐SIMS/SEM. Scientific Reports 6: e31525. 10.1038/srep31525 PMC4980676 27510918

[nph17753-bib-0003] Avanzi S , Cionini PG . 1971. A DNA cytophotometric investigation on the development of the female gametophyte of *Ginkgo biloba* . Caryologia 24: 105–116.

[nph17753-bib-0004] Barro‐Trastoy D , Dolores Gomez M , Tornero P , Perez‐Amador MA . 2020. On the way to ovules: the hormonal regulation of ovule development. Critical Reviews in Plant Sciences 39: 431–456.

[nph17753-bib-0005] Becker A , Winter KU , Meyer B , Saedler H , Theißen G . 2000. MADS‐box gene diversity in seed plants 300 million years ago. Molecular Biology and Evolution 17: 1425–1434. 11018150 10.1093/oxfordjournals.molbev.a026243

[nph17753-bib-0006] Bolger AM , Lohse M , Usadel B . 2014. Trimmomatic: a flexible trimmer for Illumina sequence data. Bioinformatics 30: 2114–2120. 24695404 10.1093/bioinformatics/btu170 PMC4103590

[nph17753-bib-0007] Breiman L . 2001. Random forests. Machine Learning 45: 5–32.

[nph17753-bib-0008] Broeckling CD , Huhman DV , Farag MA , Smith JT , May GD , Mendes P , Dixon RA , Sumner LW . 2005. Metabolic profiling of *Medicago truncatula* cell cultures reveals the effects of biotic and abiotic elicitors on metabolism. Journal of Experimental Botany 56: 323–336. 15596476 10.1093/jxb/eri058

[nph17753-bib-0009] Buchfink B , Xie C , Huson DH . 2015. Fast and sensitive protein alignment using Diamond . Nature Methods 12: 59–60. 25402007 10.1038/nmeth.3176

[nph17753-bib-0010] Carothers IE . 1907. Development of ovule and female gametophyte in *Ginkgo biloba* . Botanical Gazette 43: 116–130.

[nph17753-bib-0011] Chang S , Puryear J , Cairney J . 1993. A simple and efficient method for isolating RNA from pine trees. Plant Molecular Biology Reporter 11: 113–116.

[nph17753-bib-0012] Chen T , Cao Y , Zhang Y , Liu J , Bao Y , Wang C , Jia W , Zhao A . 2013. Random forest in clinical metabolomics for phenotypic discrimination and biomarker selection. Evidence‐Based Complementary and Alternative Medicine 2013: e298183. 10.1155/2013/298183 PMC3594909 23573122

[nph17753-bib-0013] Cheng F , Zhao B , Jiang B , Lu Y , Li W , Jin B , Wang L . 2018. Constituent analysis and proteomic evaluation of ovular secretions in *Ginkgo biloba*: not just a pollination medium. Plant Signaling and Behavior 13: e1550316. 30475662 10.1080/15592324.2018.1550316 PMC6296353

[nph17753-bib-0014] Chong J , Xia J . 2020. Using metaboanalyst 4.0 for metabolomics data analysis, interpretation, and integration with other omics data. In: Li S , ed. Computational methods and data analysis for metabolomics. New York, NY, USA: Humana Press, 337–360. 10.1007/978-1-0716-0239-3_17 31953825

[nph17753-bib-0015] Cucinotta M , Colombo L , Roig‐Villanova I . 2014. Ovule development, a new model for lateral organ formation. Frontiers in Plant Science 5: e117. 24723934 10.3389/fpls.2014.00117 PMC3973900

[nph17753-bib-0016] Douglas AW , Stevenson DW , Little DP . 2007. Ovule development in *Ginkgo biloba* L., with emphasis on the collar and nucellus. International Journal of Plant Sciences 168: 1207–1236.

[nph17753-bib-0017] Doyle JA . 2006. Seed ferns and the origin of angiosperms. The Journal of the Torrey Botanical Society 133: 169–209.

[nph17753-bib-0018] Doyle JA . 2008. Integrating molecular phylogenetic and paleobotanical evidence on origin of the flower. International Journal of Plant Sciences 169: 816–843.

[nph17753-bib-0019] Endress PK . 2011. Angiosperm ovules: diversity, development, evolution. Annals of Botany 107: 1465–1489. 21606056 10.1093/aob/mcr120 PMC3108811

[nph17753-bib-0020] Endress PK , Doyle JA . 2009. Reconstructing the ancestral angiosperm flower and its initial specializations. American Journal of Botany 96: 22–66. 21628175 10.3732/ajb.0800047

[nph17753-bib-0021] Enot DP , Beckmann M , Draper J . 2006. On the interpretation of high throughput MS based metabolomics fingerprints with random forest. In: Berthold MR , Glen RC , Fischer I , eds. International symposium on computational life science. Berlin, Heidelberg, Germany: Springer, 226–235.

[nph17753-bib-0022] Fausto C , Araniti F , Mininni AN , Crecchio C , Scagliola M , Bleve G , Dichio B , Sofo A . 2021. Differential olive grove management regulates the levels of primary metabolites in xylem sap. Plant and Soil 460: 281–296.

[nph17753-bib-0023] Feng J , Shen Y , Shi F , Li C . 2018. Embryo development, seed germination, and the kind of dormancy of *Ginkgo biloba* L. Forests 9: e700.

[nph17753-bib-0024] Figueiredo DD , Batista RA , Roszak PJ , Hennig L , Köhler C . 2016. Auxin production in the endosperm drives seed coat development in Arabidopsis. eLife 5: e20542. 27848912 10.7554/eLife.20542 PMC5135394

[nph17753-bib-0025] Figueiredo DD , Köhler C . 2014. Signalling events regulating seed coat development. Biochemical Society Transactions 42: 358–363. 24646244 10.1042/BST20130221

[nph17753-bib-0026] Figueiredo DD , Köhler C . 2018. Auxin: a molecular trigger of seed development. Genes and Development 32: 479–490. 29692356 10.1101/gad.312546.118 PMC5959232

[nph17753-bib-0027] Friedman WE . 1987. Growth and development of the male gametophyte of *Ginkgo biloba* within the ovule (*in vivo*). American Journal of Botany 74: 1797–1815.

[nph17753-bib-0028] Friedman WE , Gifford EM . 1997. Development of the male gametophyte of *Ginkgo biloba*: a window into the reproductive biology of early seed plants. In: Hori T , Ridge RW , Tulecke W , Del Tredici P , Trémouillaux‐Guiller J , Tobe H , eds. *Ginkgo biloba* a global treasure. Tokyo, Japan: Springer, 29–49.

[nph17753-bib-0029] Gasser CS , Skinner DJ . 2019. Development and evolution of the unique ovules of flowering plants. Current Topics in Developmental Biology 131: 373–399. 30612624 10.1016/bs.ctdb.2018.10.007

[nph17753-bib-0030] Gerrienne P , Meyer‐Berthaud B . 2007. The proto‐ovule *Runcaria heinzelinii* Stockmans 1968 emend. Gerrienne *et al*., 2004 (mid‐Givetian, Belgium): concept and epitypification. Review of Palaeobotany and Palynology 145: 321–323.

[nph17753-bib-0031] Gerrienne P , Meyer‐Berthaud B , Fairon‐Demaret M , Streel M , Steemans P . 2004. Runcaria, a middle devonian seed plant precursor. Science 306: 856–858. 15514154 10.1126/science.1102491

[nph17753-bib-0032] Guan C , Cen HF , Cui X , Tian DY , Tadesse D , Zhang YW . 2019. Proline improves switchgrass growth and development by reduced lignin biosynthesis. Scientific Reports 9: e20117. 10.1038/s41598-019-56575-9 PMC6934488 31882839

[nph17753-bib-0033] Guan R , Zhao Y , Zhang HE , Fan G , Liu X , Zhou W , Shi C , Wang J , Liu W , Liang X *et al*. 2016. Draft genome of the living fossil *Ginkgo biloba* . Gigascience 5: e49. 27871309 10.1186/s13742-016-0154-1 PMC5118899

[nph17753-bib-0034] Hammes UZ , Nielsen E , Honaas LA , Taylor CG , Schachtman DP . 2006. AtCAT6, a sink‐tissue‐localized transporter for essential amino acids in Arabidopsis. The Plant Journal 48: 414–426. 17052324 10.1111/j.1365-313X.2006.02880.x

[nph17753-bib-0035] Horai H , Arita M , Kanaya S , Nihei Y , Ikeda T , Suwa K , Ojima Y , Tanaka K , Tanaka S , Aoshima K *et al*. 2010. MassBank: a public repository for sharing mass spectral data for life sciences. Journal of Mass Spectrometry 45: 703–714. 20623627 10.1002/jms.1777

[nph17753-bib-0036] Jin B , Jiang X , Wang D , Zhang L , Wan Y , Wang L . 2012a. The behavior of pollination drop secretion in *Ginkgo biloba* L. Plant Signaling and Behavior 7: 1168–1176. 22899081 10.4161/psb.21122 PMC3489653

[nph17753-bib-0037] Jin B , Wang D , Lu Y , Jiang XX , Zhang M , Zhang L , Wang L . 2012b. Female short shoot and ovule development in *Ginkgo biloba* L. with emphasis on structures associated with wind pollination. ISRN Botany 2012: e230685.

[nph17753-bib-0038] Kishor PBK , Sangam S , Amrutha RN , Laxmi PS , Naidu KR , Rao KRSS , Rao S , Reddy KJ , Theriappan P , Sreenivasulu N . 2005. Regulation of proline biosynthesis, degradation, uptake and transport in higher plants: its implications in plant growth and abiotic stress tolerance. Current Science 88: 424–438.

[nph17753-bib-0039] Kupke T , Hernández‐Acosta P , Culiáñez‐Macià FA . 2003. 4′‐Phosphopantetheine and coenzyme A biosynthesis in plants. Journal of Biological Chemistry 278: 38229–38237. 12860978 10.1074/jbc.M306321200

[nph17753-bib-0040] Landi M , Araniti F , Flamini G , Piccolo EL , Trivellini A , Abenavoli MR , Guidi L . 2020. “Help is in the air”: volatiles from salt‐stressed plants increase the reproductive success of receivers under salinity. Planta 251: e48. 10.1007/s00425-020-03344-y 31932951

[nph17753-bib-0041] Lee CL . 1955. Fertilization in *Ginkgo biloba* . Botanical Gazette 117: 79–100.

[nph17753-bib-0042] Lisec J , Schauer N , Kopka J , Willmitzer L , Fernie AR . 2006. Gas chromatography mass spectrometry‐based metabolite profiling in plants. Nature Protocols 1: 387–396. 17406261 10.1038/nprot.2006.59

[nph17753-bib-0043] Livak KJ , Schmittgen TD . 2001. Analysis of relative gene expression data using real‐time quantitative PCR and the 2^−ΔΔCT^ method. Methods 25: 402–408. 11846609 10.1006/meth.2001.1262

[nph17753-bib-0044] Love MI , Huber W , Anders S . 2014. Moderated estimation of fold change and dispersion for RNA‐seq data with DESeq2. Genome Biology 15: e550. 10.1186/s13059-014-0550-8 PMC4302049 25516281

[nph17753-bib-0045] Lu Z , Jiang B , Zhao B , Mao X , Lu J , Jin B , Wang L . 2020. Liquid profiling in plants: identification and analysis of extracellular metabolites and miRNAs in pollination drops of *Ginkgo biloba* . Tree Physiology 40: 1420–1436. 32542386 10.1093/treephys/tpaa073

[nph17753-bib-0046] Meade LE , Plackett AR , Hilton J . 2020. Reconstructing development of the earliest seed integuments raises a new hypothesis for the evolution of ancestral seed‐bearing structures. New Phytologist 229: 1782–1794. 32639670 10.1111/nph.16792

[nph17753-bib-0047] Münch E . 1930. Die stoffbewegungen in der pflanze. Jena, Germany: Fischer.

[nph17753-bib-0048] Nepi M . 2014. Beyond nectar sweetness: the hidden ecological role of non‐protein amino acids in nectar. Journal of Ecology 102: 108–115.

[nph17753-bib-0049] Nepi M , Little S , Guarnieri M , Nocentini D , Prior N , Gill J , Barry Tomlinson P , Ickert‐Bond SM , Pirone C , Pacini E *et al*. 2017. Phylogenetic and functional signals in gymnosperm ovular secretions. Annals of Botany 120: 923–936. 29045531 10.1093/aob/mcx103 PMC5710648

[nph17753-bib-0050] Nigris S , D’Apice G , Moschin S , Ciarle R , Baldan B . 2021. Fleshy structures associated with ovule protection and seed dispersal in gymnosperms: a systematic and evolutionary overview. Critical Reviews in Plant Sciences 40: 285–302.

[nph17753-bib-0051] Patro R , Duggal G , Love MI , Irizarry RA , Kingsford C . 2017. Salmon provides fast and bias‐aware quantification of transcript expression. Nature Methods 14: 417–419. 28263959 10.1038/nmeth.4197 PMC5600148

[nph17753-bib-0052] Percival B , Gibson M , Leenders J , Wilson PB , Grootveld M . 2020. Univariate and multivariate statistical approaches to the analysis and interpretation of NMR‐based metabolomics datasets of increasing complexity. In: Wilson PB , Grootveld M , eds. Computational techniques for analytical chemistry and bioanalysis. London, UK: Royal Society of Chemistry, 1–40. doi: 10.1039/9781788015882-00001.

[nph17753-bib-0053] Pfaffl MW . 2001. A new mathematical model for relative quantification in real‐time RT‐PCR. Nucleic Acids Research 29: e45. 11328886 10.1093/nar/29.9.e45 PMC55695

[nph17753-bib-0054] Prestianni C , Gerrienne P . 2010. Early seed plant radiation: an ecological hypothesis. Geological Society, London, Special Publications 339: 71–80.

[nph17753-bib-0055] Prior N , Little SA , Boyes I , Griffith P , Husby C , Pirone‐Davies C , Stevenson DW , Tomlinson PB , von Aderkas P . 2019. Complex reproductive secretions occur in all extant gymnosperm lineages: a proteomic survey of gymnosperm pollination drops. Plant Reproduction 32: 153–166. 30430247 10.1007/s00497-018-0348-z PMC6500509

[nph17753-bib-0056] Saccenti E , Hoefsloot HC , Smilde AK , Westerhuis JA , Hendriks MM . 2014. Reflections on univariate and multivariate analysis of metabolomics data. Metabolomics 10: 361–374.

[nph17753-bib-0057] Sansone SA , Fan T , Goodacre R , Griffin JL , Hardy NW , Kaddurah‐Daouk R , Kristal BS , Lindon J , Mendes P , Morrison N *et al*. 2007. The metabolomics standards initiative. Nature Biotechnology 25: 846–849. 10.1038/nbt0807-846b 17687353

[nph17753-bib-0058] Serdyuk OP , Smolygina LD , Ivanova EP , Firsov AP , Pogrebnoi PV . 2000. 4‐Hydroxyphenethyl alcohol – a new cytokinin‐like substance isolated from phototrophic bacterium *Rhodospirillum rubrum*. Exhibition of activity on plants and transformed mammalian cells. Process Biochemistry 36: 475–479.

[nph17753-bib-0059] Serdyuk OP , Smolygina LD , Muzafarov EN , Adanin VM , Arinbasarov MU . 1995. 4‐Hydroxyphenethyl alcohol – a new cytokinin‐like substance from the phototrophic purple bacterium *Rhodospirillum rubrum* 1R. FEBS Letters 365: 10–12. 7539770 10.1016/0014-5793(95)00430-h

[nph17753-bib-0060] Shivanna KR . 2003. Pollen biology and biotechnology. Enfield, NH, USA: CRC Press.

[nph17753-bib-0061] Singh B , Kaur P , Singh RD , Ahuja PS . 2008. Biology and chemistry of *Ginkgo biloba* . Fitoterapia 79: 401–418. 18639617 10.1016/j.fitote.2008.05.007

[nph17753-bib-0062] Singh H . 1978. Embryology of gymnosperms. Berlin, Germany: Gerbrüder Borntraeger.

[nph17753-bib-0063] Sprecher A . 1907. Le *Ginkgo biloba* . PhD thesis, University of Geneva, Geneva, Switzerland.

[nph17753-bib-0064] Sturm A , Tang GQ . 1999. The sucrose‐cleaving enzymes of plants are crucial for development, growth and carbon partitioning. Trends in Plant Science 4: 401–407. 10498964 10.1016/s1360-1385(99)01470-3

[nph17753-bib-0065] Takaso T . 1980. Developmental study of the integument in gymnosperms. 1. *Ginkgo biloba* L. The Journal of Japanese Botany 55: 14–27.

[nph17753-bib-0066] Terashima N , Ko C , Matsushita Y , Westermark U . 2016. Monolignol glucosides as intermediate compounds in lignin biosynthesis. Revisiting the cell wall lignification and new ^13^C‐tracer experiments with *Ginkgo biloba* and *Magnolia liliiflora* . Holzforschung 70: 801–810.

[nph17753-bib-0067] Verbruggen N , Hermans C . 2008. Proline accumulation in plants: a review. Amino Acids 35: 753–759. 18379856 10.1007/s00726-008-0061-6

[nph17753-bib-0068] Volpi e Silva N , Mazzafera P , Cesarino I . 2019. Should I stay or should I go: are chlorogenic acids mobilized towards lignin biosynthesis? Phytochemistry 166: e112063. 31280091 10.1016/j.phytochem.2019.112063

[nph17753-bib-0069] Wang D , Lu Y , Zhang M , Lu Z , Luo K , Cheng F , Wang L . 2014. Structure and function of the neck cell during fertilization in *Ginkgo biloba* L. Trees 28: 995–1005.

[nph17753-bib-0070] White WH , Gunyuzlu PL , Toyn JH . 2001. *Saccharomyces cerevisiae* is capable of *de novo* pantothenic acid biosynthesis involving a novel pathway of β‐alanine production from spermine. Journal of Biological Chemistry 276: 10794–10800. 11154694 10.1074/jbc.M009804200

[nph17753-bib-0071] Xia J , Wishart DS . 2011. Web‐based inference of biological patterns, functions and pathways from metabolomic data using MetaboAnalyst . Nature Protocols 6: 743–760. 21637195 10.1038/nprot.2011.319

[nph17753-bib-0072] Yamauchi K , Yasuda S , Fukushima K . 2002. Evidence for the biosynthetic pathway from sinapic acid to syringyl lignin using labeled sinapic acid with stable isotope at both methoxy groups in *Robinia pseudoacacia* and *Nerium indicum* . Journal of Agricultural and Food Chemistry 50: 3222–3227. 12009990 10.1021/jf011565x

[nph17753-bib-0073] Zhao Q , Dixon RA . 2011. Transcriptional networks for lignin biosynthesis: more complex than we thought? Trends in Plant Science 16: 227–233. 21227733 10.1016/j.tplants.2010.12.005

