# Supplementary material for: The role of pollination in controlling Ginkgo biloba ovule development
Source: New Phytol. 2021 Oct 11;232(6):2353–68. doi: 10.1111/nph.17753 (PMC9292720; doi:10.1111/nph.17753)
Supplement: Supplementary file 1 — Fig. S1 Ginkgo biloba ovule development: supporting morphological analysis. Fig. S2 Correlation analysis between the two RNA‐seq experiments performed on the two plants of Ginkgo biloba (GA and GN) in the five sequenced stages. Fig. S3 Comparison of the expression levels (log2 TPM) of selected genes in the two plants of Ginkgo biloba. Fig. S4 Gene Ontology (GO) enrichment bar chart. Fig. S5 Kyoto Encyclopedia of Genes and Genomes (KEGG) enrichment bar chart. Fig. S6 Comparison between the RNA‐seq expression levels (blue line) and RT‐qPCR expression levels (orange line) performed on Ginkgo biloba ovules during the pollination phase. Notes S1 Deepening of univariate and multivariate approach in metabolomics experiments. Table S2 Primer sequences used to amplify Ginkgo biloba selected genes. [file NPH-232-2353-s003.pdf]

***New Phytologist* Supporting Information**

Article title: **The role of pollination in controlling *Ginkgo biloba* ovule development**

Authors: Greta D'Apice*[1,2], Silvia Moschin*[1,2], Fabrizio Araniti[3], Sebastiano Nigris[1,2], Maurizio Di Marzo[4], Antonella Muto[5], Camilla Banfi[4], Leonardo Bruno[5], Lucia Colombo[4], Barbara Baldan[1,2]

[1]Botanical Garden, University of Padova, 25123 Padova, Italy; [2]Department of Biology, University of Padova, 35121 Padova, Italy; [3]Department of Agricultural and Environmental Sciences, University of Milano, 20133 Milano, Italy; [4]Department of Biosciences, University of Milano, 20133 Milano, Italy; [5]Department of Biology, Ecology and Earth Sciences (DiBEST), University of Calabria, 87036 Arcavacata of Rende (CS), Italy

Article acceptance date: 13 September 2021

The following Supporting Information is available for this article:

Fig. S1 *Ginkgo biloba* ovule development: supporting morphological analysis.

Fig. S2 Correlation analysis between the two RNA-seq experiments performed on the two plants of *Ginkgo biloba* (GA and GN) in the five sequenced stages.

Fig. S3 Comparison of the expression levels (Log2 TPM) of selected genes in the two plants of *Ginkgo biloba*.

Fig. S4 Gene Ontology (GO) enrichment bar chart.

Fig. S5 Kyoto Encyclopedia of Genes and Genomes (KEGG) enrichment bar chart.

Fig. S6 Comparison between the RNA-seq expression levels (blue line) and RT-qPCR expression levels (orange line) performed on *Ginkgo biloba* ovules during the pollination phase.

Table S1 *Ginkgo biloba* genome annotation.

Table S2 Primer sequences used to amplify *Ginkgo biloba* selected genes.

Table S3 *Ginkgo biloba* orthologous genes of Arabidopsis 'switch genes'.

Table S4 GC-MS-driven untargeted metabolomics analysis data.

Notes S1 Deepening of univariate and multivariate approach in metabolomics experiments.

Fig. S1 *Ginkgo biloba* ovule development: supporting morphological analysis. a) STAGE 4: Integument growth begins to enclose the nucellus. Longitudinal section of a paraffin-embedded ovule within bud, showing the nucellus and the flanking integument. b) STAGE 7: Pre-pollination stage. Micropyle, micropyle canal, and pollen chamber are completely formed. Longitudinal section of a paraffin-embedded ovule showing the completely formed pollen chamber connected with the micropyle. c) STAGE 8: Pollination drop stage. Longitudinal section of a paraffin-embedded ovule in which the tapetal cells encircling the female gametophyte are marked with black arrow-head. d) STAGE 9: Female gametophyte growing; integument layers are becoming distinguishable. Longitudinal section of a paraffin-embedded ovule, in which the germinated pollen grain is visible within the pollen chamber (black arrow-head). e) STAGE 9: Female gametophyte growing; integument layers are becoming distinguishable. Longitudinal section of a paraffin-embedded ovule, in which the three layers of the single integument are distinguishable. f) STAGE 9: Female gametophyte growing; integument layers are becoming distinguishable. Longitudinal section of a paraffin-embedded ovule in which the tapetal cells encircling the female gametophyte are degenerating (black arrow-head). g) STAGE 12: Archegonia completely formed. Longitudinal section of a paraffin-embedded ovule in which archegonia and the tent pole between them are visible. *e = endotesta;* Fg= female gametophyte; M = micropyle; N = nucellus; Pc = pollen chamber; Pg = pollen grain; *sar = sarcotesta*; *scl = sclerotesta*; Ta = tapetum; Tp = tent pole. Scale bars: a, b, c, d = 200 µm; e, f, g = 500 µm.

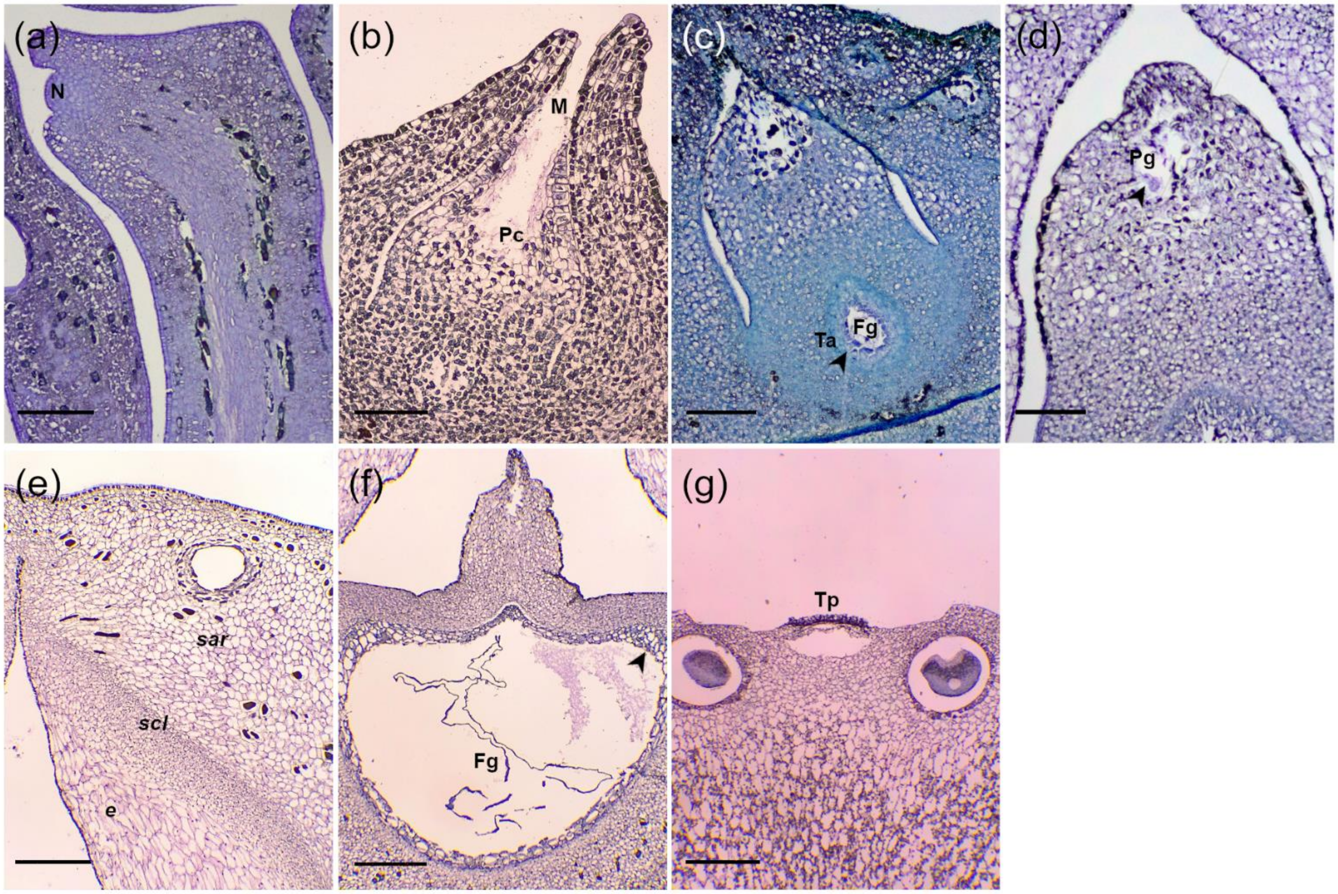

(a)
N
(b)
M
Pc
(c)
Fg
Ta
(d)
Pg
(e)
sar
scl
e
(f)
Fg
(g)
Tp

Fig. S2 Correlation analysis between the two RNA-seq experiments performed on the two plants of *Ginkgo biloba* (GA and GN) in the five sequenced stages. The two plants were used as biological replicates.

sample	GA 7	GA 8.1	GA 8.2	GA 8.3	GA 8.4	GN 7	GN 8.1	GN 8.2	GN 8.3	GN 8.4
GA 7	1.00	0.91	0.92	0.92	0.82	0.99	0.91	0.92	0.86	0.78
GA 8.1	0.91	1.00	0.99	0.98	0.85	0.92	0.95	0.98	0.98	0.80
GA 8.2	0.92	0.99	1.00	0.99	0.88	0.94	0.96	0.99	0.97	0.83
GA 8.3	0.92	0.98	0.99	1.00	0.91	0.93	0.97	0.98	0.97	0.87
GA 8.4	0.82	0.85	0.88	0.91	1.00	0.83	0.95	0.88	0.88	0.99
GN 7	0.99	0.92	0.94	0.93	0.83	1.00	0.93	0.93	0.88	0.79
GN 8.1	0.91	0.95	0.96	0.97	0.95	0.93	1.00	0.97	0.93	0.93
GN 8.2	0.92	0.98	0.99	0.98	0.88	0.93	0.97	1.00	0.98	0.84
GN 8.3	0.86	0.98	0.97	0.97	0.88	0.88	0.93	0.98	1.00	0.80
GN 8.4	0.78	0.80	0.83	0.87	0.99	0.79	0.93	0.84	0.80	1.00

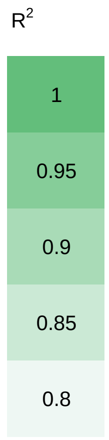

Fig. S3 Comparison of the expression levels (Log2 TPM) of selected genes in the two plants of *Ginkgo biloba*. The two plants (GA and GN) were treated as two biological replicates: GA (orange line) and GN (blue line). X axis indicates the five developmental stages of *Ginkgo* ovules, Y axis indicates the gene expression level calculated as Transcripts Per kilobase per Million of reads (TPM).

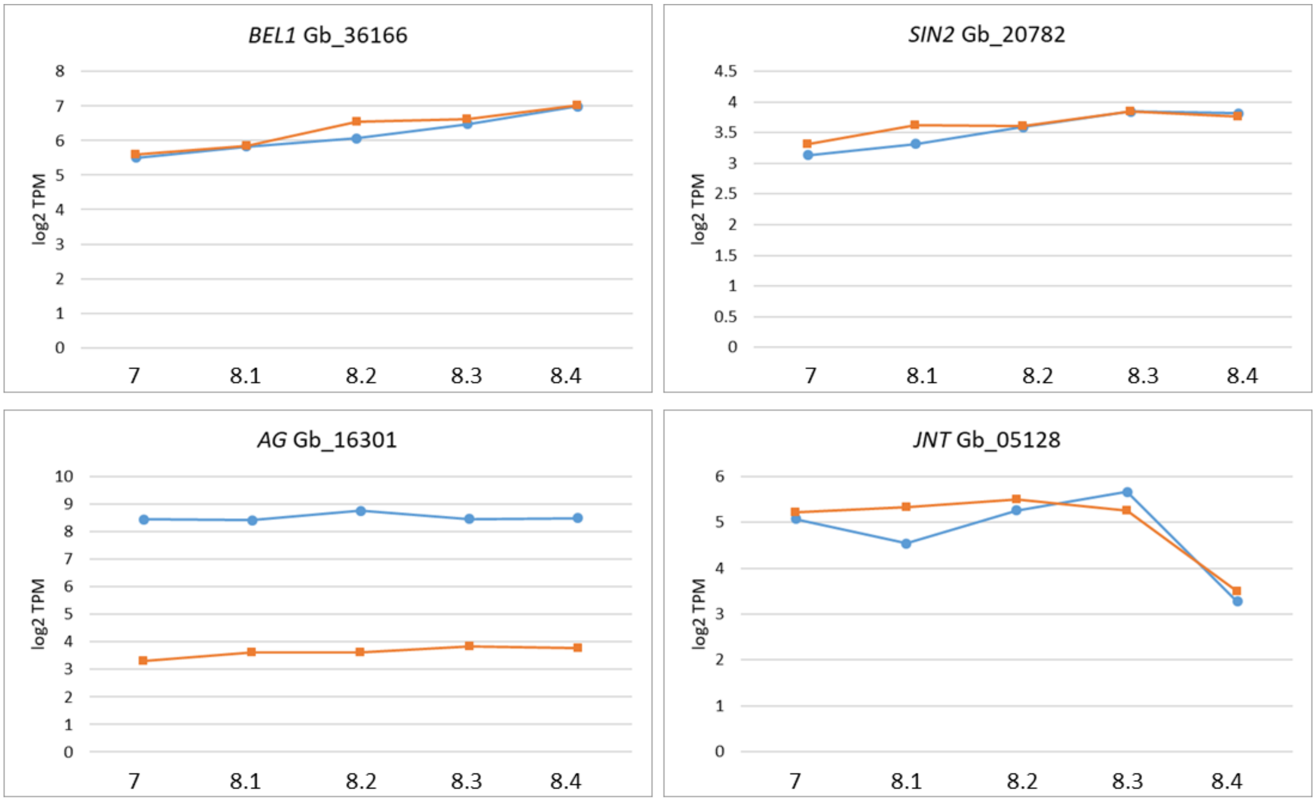

Fig. S4 Gene Ontology (GO) enrichment bar chart. Comparison between the five sequenced stages of *Ginkgo biloba* ovules during the pollination phase.

* Significantly enriched pathway

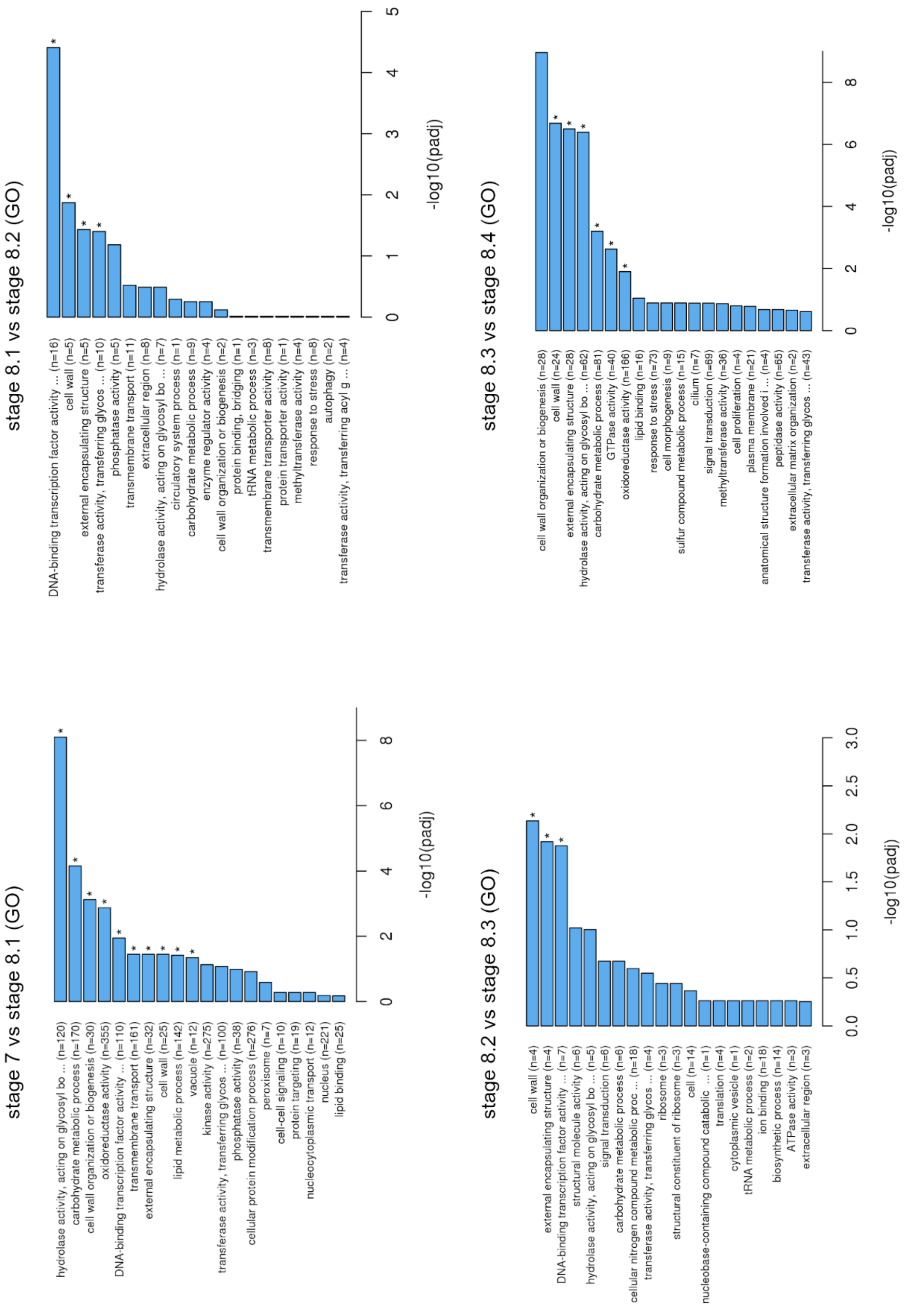

Fig. S5 Kyoto Encyclopedia of Genes and Genomes (KEGG) enrichment bar chart. Comparison between the five sequenced stages of *Ginkgo biloba* ovules during the pollination phase.

* Significantly enriched pathway

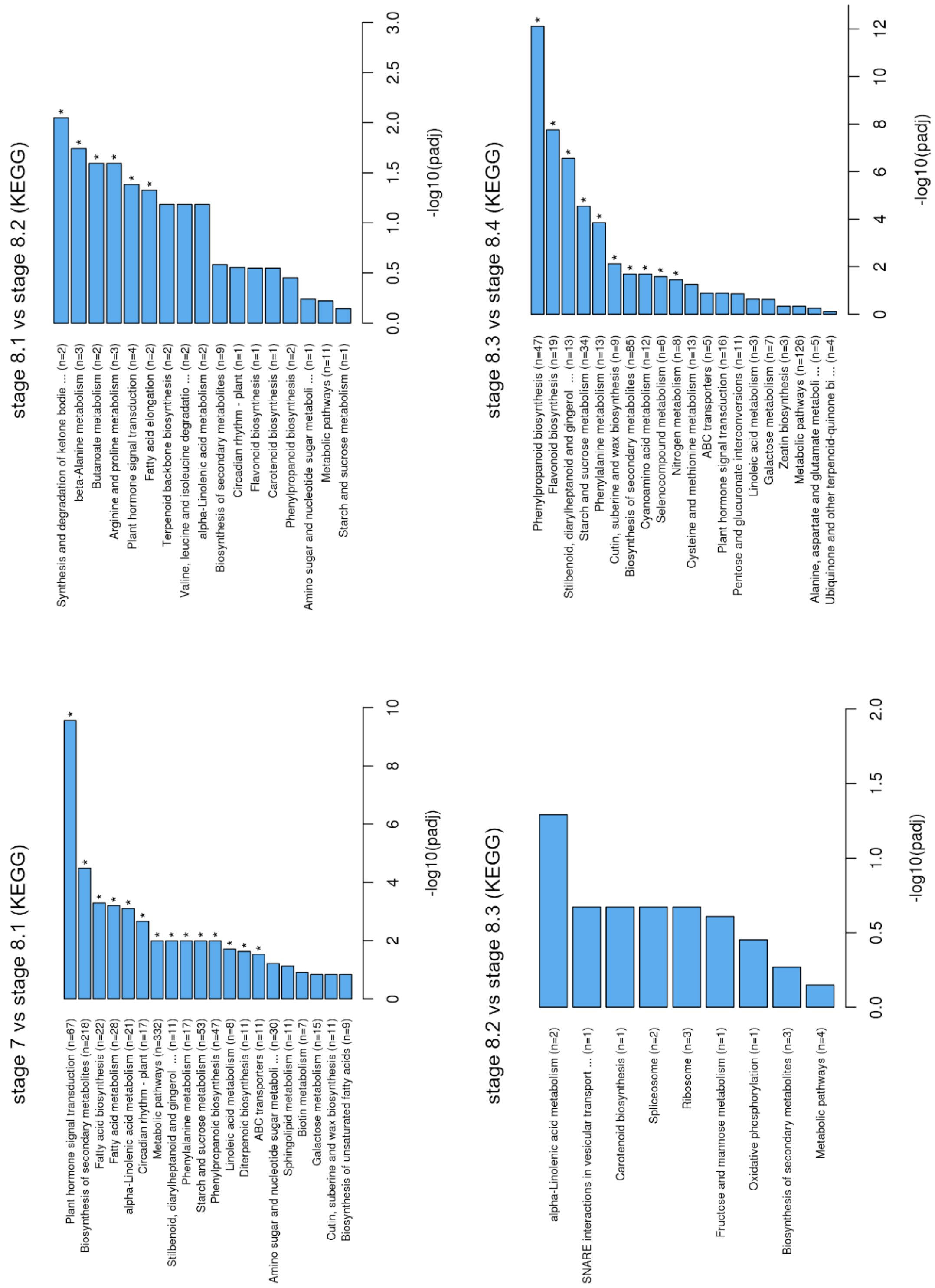

Fig. S6 Comparison between the RNA-seq expression levels (blue line) and RT-qPCR expression levels (orange line) performed on *Ginkgo biloba* ovules during the pollination phase. X axis indicates the five developmental stages of *Ginkgo* ovules, Y axis indicates the expression levels of the Gene Of Interest (GOI) normalized at the expression level of the internal reference gene *EF2* (TPM GOI/TPM *EF2*). Error bars represent SE of the means.

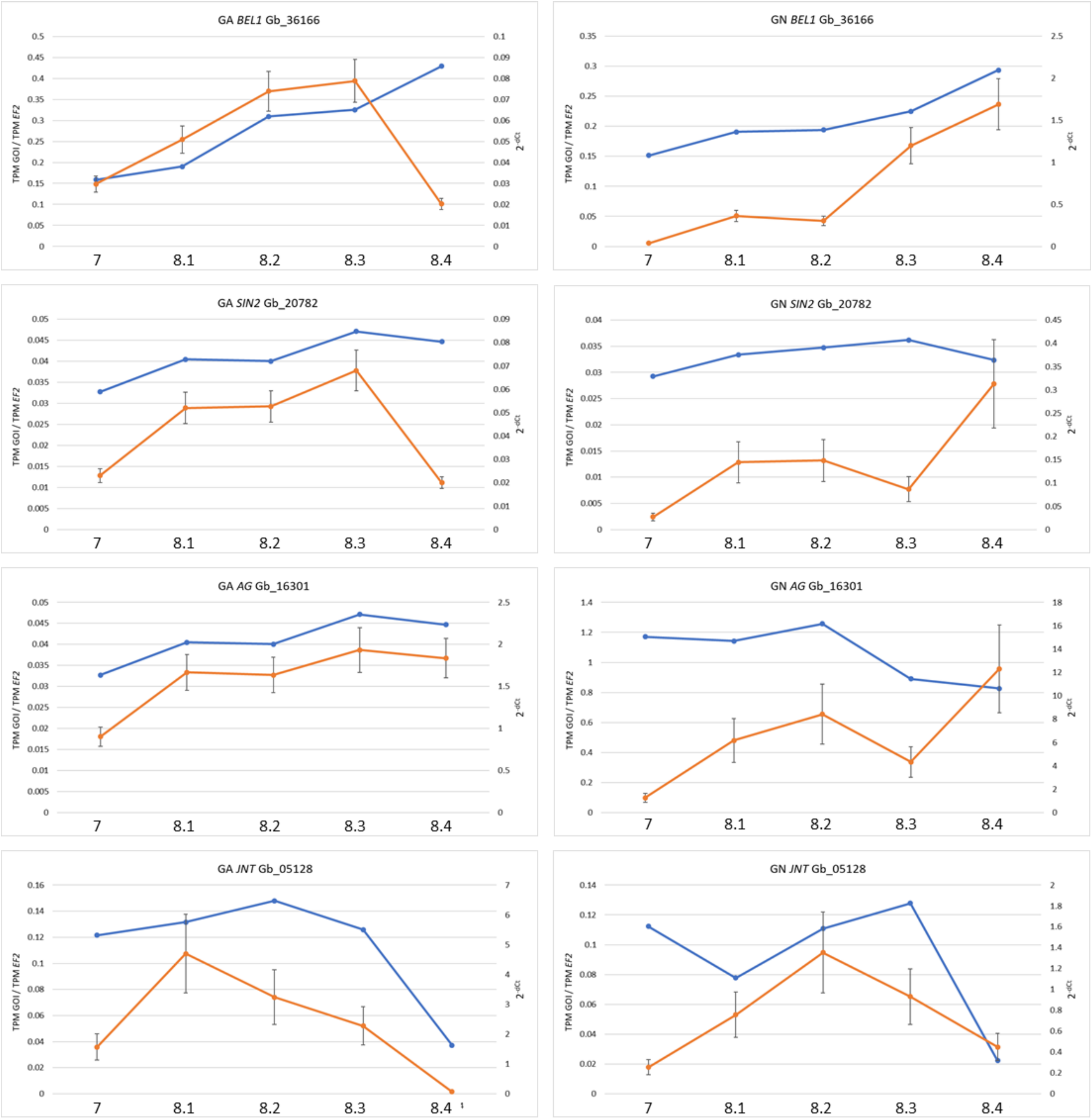

Table S1 *Ginkgo biloba* genome annotation. Table S1 is available as a separated file.

Table S2 Primer sequences used to amplify *Ginkgo biloba* selected genes.

Gingko CDS	Gene	Arabidopsis orthologous gene	Primer Name	Primer
Gb_16301	*GBM5*	*AGAMOUS*	AG_For	CCAGTTTCTGCGCACTAAGA
			AG_Rev	TCATCCAAGTTGAAGGGCC
Gb_05128	*GbMADS5*	*SVP/JNT*	JNT_For	TGCGGTCGACGAAGATTCTG
			JNT_Rev	TCAACTTTGGCGCAAGTGAAC
Gb_36166		*BEL1*	2_BEL1_For	GAGTTGGTGCAACAGAATCC
			2_BEL1_Rev	CCAGCATAACCTGGGTTCAT
Gb_20782		*SIN2*	SIN2_For	GCTAAATACCCGCTCTGCA
			SIN2_Rev	GCATGATCCTGTGTGTGATC
Gb_02896		*EF2*	EF2_For	TCCATCTTCCTTCTCCATCC
			EF2_Rev	CTTACCTTCATACCTGTTGCC

Table S3 *Ginkgo biloba* orthologous genes of Arabidopsis 'switch genes'* are in most of the cases not differentially expressed between stages 7 and 8.4, and between sub-stages 8.1 and 8.4 of ovule development. Table S3 is available as a separated file.

*Arabidopsis 'switch genes' are the genes activated upon fertilization that are required to activate the developmental programs that lead the transformation of the ovule integument into the seed coat.

Table S4 Data of the GC-MS-driven untargeted metabolomics experiments conducted on *Ginkgo biloba* ovules during the pollination phase. Table S4 is available as a separated file.

Notes S1 Deepening of univariate and multivariate approach in metabolomics experiments.

It is well stated that untargeted-metabolomics studies are designed to obtain a large quantity of data concerning the abundance of large numbers of metabolites in biological material. During the collection of such large data sets, it is assumed that the (co-)variation of the metabolite abundances contains the information necessary in understanding the biological phenomenon under study. Anyway, among all the significantly altered metabolites, only a subset is often related to the phenomenon. Therefore, the challenge of the statistical and bioinformatic approach is to extract from this large dataset those metabolites of interest and exclude those whose variation is not of interest for the goals of the research. In this context, the joint application of both univariate and multivariate analysis is routinely used to extract relevant information from the data to provide biological knowledge on the phenomenon under observation (for a detailed review on the joint application of univariate and multivariate analysis, see Saccenti *et al*., 2014 and Percival *et al*., 2020). In particular, in the present study, we used both unsupervised Principal Component Analysis (PCA) (a dimension reduction methodology applied without considering the correlation between the dependent variable and the independent variables, which is useful for data mining and outlier detection) and the supervised Partial Least Square - Discriminant Analysis (PLS-DA) (a versatile algorithm that can be used for predictive and descriptive modelling as well as for discriminative variable selection). Data were successively analysed through one way ANOVA to highlight statistically significant features among all the metabolites annotated and then through the machine learning approach Random forest. The latter is a machine learning technique, which establishes the outcome based on the predictions of the decision trees, used to solve regression and classification problems, combining many classifiers to solve complex problems. This algorithm is routinely used in metabolomics to extract, from a large dataset, variables (potential biomarkers of a given phenomenon) that best classify the data into different groups, since compared to other classification methods, it has been shown to perform better on metabolomics data for phenotypic classification and biomarker selection (Breiman, 2001; Enot *et al*., 2006; Chen *et al*., 2013).

Finally, data were analysed through enrichment analysis (a method designed to help in identifying and biologically interpret patterns of metabolite concentration changes in a given pathway) and pathway analysis (using the Metaboanalyst tool MetPA). Unlike the enrichment analysis, which only considers the changes in concentration of metabolites belonging to a specific pathway, the pathway analysis also considers the topology of the metabolites and their importance in the

pathway. This allows establishing how a change in concentration of a specific metabolite/s could impact the functionality of a specific pathway inhibiting or bursting it (Xia & Wishart, 2011).

References

Breiman L. 2001. Random forests. *Machine learning* **45**: 5-32.

Chen T, Cao Y, Zhang Y, Liu J, Bao Y, Wang C, Jia W, Zhao A. 2013. Random forest in clinical metabolomics for phenotypic discrimination and biomarker selection. *Evidence-Based complementary and alternative Medicine* **2013**: 1-11.

Enot DP, Beckmann M, Draper J. 2006. On the interpretation of high throughput MS based metabolomics fingerprints with random forest. In Berthold MR, Glen RC, Fischer I, eds. *International Symposium on Computational Life Science*. Springer, Berlin, Heidelberg, 226-235.

Percival B, Gibson M, Leenders J, Wilson PB, Grootveld M. 2020. Univariate and Multivariate Statistical Approaches to the Analysis and Interpretation of NMR-based Metabolomics Datasets of Increasing Complexity. In Wilson PB, Grootveld M, eds. *Computational Techniques for Analytical Chemistry and Bioanalysis*. London, UK: Royal Society of Chemistry, 1-40. doi: 10.1039/9781788015882-00001.

Saccenti E, Hoefsloot HC, Smilde AK, Westerhuis JA, Hendriks MM. 2014. Reflections on univariate and multivariate analysis of metabolomics data. *Metabolomics* **10:** 361-374.

Xia J, Wishart DS. 2011. Web-based inference of biological patterns, functions and pathways from metabolomic data using MetaboAnalyst. *Nature protocols* **6**: 743-760.
